# Prediction and analysis of nucleosome exclusion regions in the human genome

**DOI:** 10.1186/1471-2164-9-186

**Published:** 2008-04-22

**Authors:** Ahmed Radwan, Akmal Younis, Peter Luykx, Sawsan Khuri

**Affiliations:** 1 Department of Electrical and Computer Engineering, University of Miami College of Engineering, Coral Gables, FL 33124, USA; 2 Department of Biology, University of Miami, Coral Gables, FL 33124, USA; 3 Center for Computational Science, and the Dr. John T. Macdonald Foundation Department of Human Genetics and Genomic Medicine, University of Miami Miller School of Medicine, Miami, FL 33136 USA

## Abstract

**Background:**

Nucleosomes are the basic structural units of eukaryotic chromatin, and they play a significant role in regulating gene expression. Specific DNA sequence patterns are known, from empirical and theoretical studies, to influence DNA bending and flexibility, and have been shown to exclude nucleosomes. A whole genome localization of these patterns, and their analysis, can add important insights on the gene regulation mechanisms that depend upon the structure of chromatin in and around a gene.

**Results:**

A whole genome annotation for nucleosome exclusion regions (NXRegions) was carried out on the human genome. Nucleosome exclusion scores (NXScores) were calculated individually for each nucleotide, giving a measure of how likely a specific nucleotide and its immediate neighborhood would impair DNA bending and, consequently, exclude nucleosomes. The resulting annotations were correlated with 19055 gene expression profiles. We developed a new method based on Grubbs' outliers test for ranking genes based on their tissue specificity, and correlated this ranking with NXScores. The results show a strong correlation between tissue specificity of a gene and the propensity of its promoter to exclude nucleosomes (the promoter region was taken as -1500 to +500 bp from the RefSeq-annotated transcription start site). In addition, NXScores correlated well with gene density, gene expression levels, and DNaseI hypersensitive sites.

**Conclusion:**

We present, for the first time, a whole genome prediction of nucleosome exclusion regions for the human genome (the data are available for download from Additional Materials). Nucleosome exclusion patterns are correlated with various factors that regulate gene expression, which emphasizes the need to include chromatin structural parameters in experimental analysis of gene expression.

## Background

Nucleosomes are DNA-protein complexes that form the building blocks of eukaryotic chromatin. They are involved in genome condensation, and play a major role in the regulation of gene expression [1]. Each nucleosome is made up of eight histone proteins that together form a structural unit able to accommodate 147 base pairs of DNA wound around it. The DNA sequence has to have the flexibility and curvature that allows it to circle around a nucleosome [2]. Empirical and theoretical studies have both shown that there are certain DNA sequence patterns that are too rigid to form such loops [3]. These patterns include GC-rich motifs as well as poly-A and poly-T tracts, and we have previously compiled them into NXSensor, a web tool that predicts which DNA sequences would not be conducive to nucleosome binding; we called these motifs *nucleosome exclusion sequences *[4].

Transcriptional regulation in eukaryotes is a complex process, as exemplified by recent publications [5,6]. The formation and positioning of nucleosomes are crucial steps in gene regulation, in that they influence access to DNA by the transcriptional machinery. Experimental work on nucleosome positioning in yeast [7-11] and fly [12] has yielded significant results, and technological progress is such that we are quickly learning more about nucleosome positioning in the human genome [13,14].

Experimental work that verifies where a nucleosome is positioned is dependent upon when the cells were sampled, and on which tissue or cell line the analysis was carried out. In addition, it is known that nucleosomes slide to allow certain regulatory mechanisms to take place [15], and it has been shown in yeast that nucleosomes are only occasionally positioned by intrinsic sequence signals [16]. We therefore chose nucleosome exclusion sequences as our predictive method, rather than nucleosome positioning sequences. We can with a certain level of certainty predict where nucleosomes would not bind, and it is therefore inferred that they can, and probably do, bind elsewhere.

Reported studies in [4,17] observed certain trends in the nucleosome exclusion patterns of promoter regions. Both studies showed that there is a peak of nucleosome exclusion sequences just upstream of the transcriptional start site of genes. This pattern has subsequently been found to be true also in yeast [9]. The studies in [4,17] found that widely expressed genes, sometimes referred to as housekeeping genes, had a higher nucleosome exclusion potential than did tissue specific genes. This implies that the promoter regions of widely expressed genes were less likely to have nucleosomes in them than were the promoter regions of genes that had a narrow tissue distribution. This may allow easy access of the transcriptional machinery to the DNA of ubiquitously expressed genes. However, these studies had taken relatively small numbers of carefully selected human genes: 100 of each category in the case of [4], and 500 each in [17], and they both relied on manual selection and categorization of genes. The question remained whether there is a genome-wide trend of a gradient of nucleosome positioning potentials, and what implications this may have for the specificity of gene expression. These were the initial questions that we set out to answer in this study.

The objective of the present study, therefore, was to carry out a whole genome annotation of nucleosome exclusion regions (NXRs) in the human genome, and to correlate the results with tissue specificity, gene expression levels, and DNaseI hypersensitive sites. We calculated nucleosome exclusion scores (NXScores) across the whole genome, and observed NXScore trends in promoter regions. We classified tissue specific and widely expressed genes according to a new method proposed here based on Grubbs' outliers test, and validated the results using a previously described method based on Shannon's entropy [18]. From a computational perspective, patterns such as NXRs and NXSs are fuzzy, non-exact, and overlapping, which poses a challenge for the analysis of all 3.4 billion base pairs of the whole human genome. We therefore developed a pilot grid architecture that can carry out such computationally intensive tasks. In this paper we report our results in the context of the regulation of gene expression.

## Results and Discussion

### Nucleosome Exclusion Landscape

**Figure 1:**
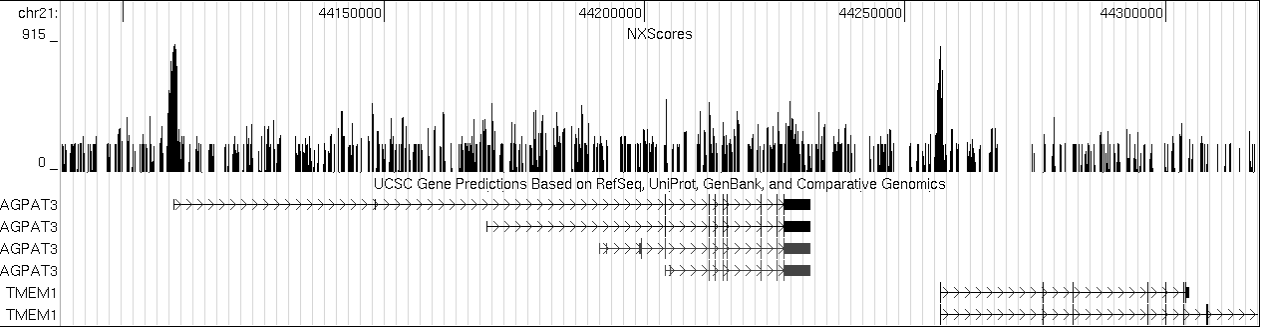
**NXScore peaks around TSSs**. An example of NXScore peaks around the transcriptional start site of genes. Shown above are the NXScores for two neighboring genes on chromosome 21. The figure was prepared by uploading NXScore results as a Custom Track on the UCSC Genome Browser, and taking a snapshot with the Known Genes track.

First we constructed a whole genome landscape of nucleosome exclusion regions and calculated their exclusion scores. The results were compiled as GFF [19] and Wiggle [20] files for each of the human chromosomes, and are made available in Additional files 1, 2, 3, 4, 5 &6. This data is being made publicly available by the UCSC Genome Browser under their Custom Tracks Page [21]. Immediately obvious from the data is the fact that NXScores increase significantly at and around the transcriptional start sites (TSSs) of genes (Figure 1). This confirms previous observations that, regardless of how many nucleosomes there may be in a given promoter region, nucleosomes are preferentially excluded from the immediate area where the transcriptional machinery needs easy access to the DNA [4]. The sections below highlight other observations and correlations we found.

### Correlation with Gene Density

**Figure 2:**
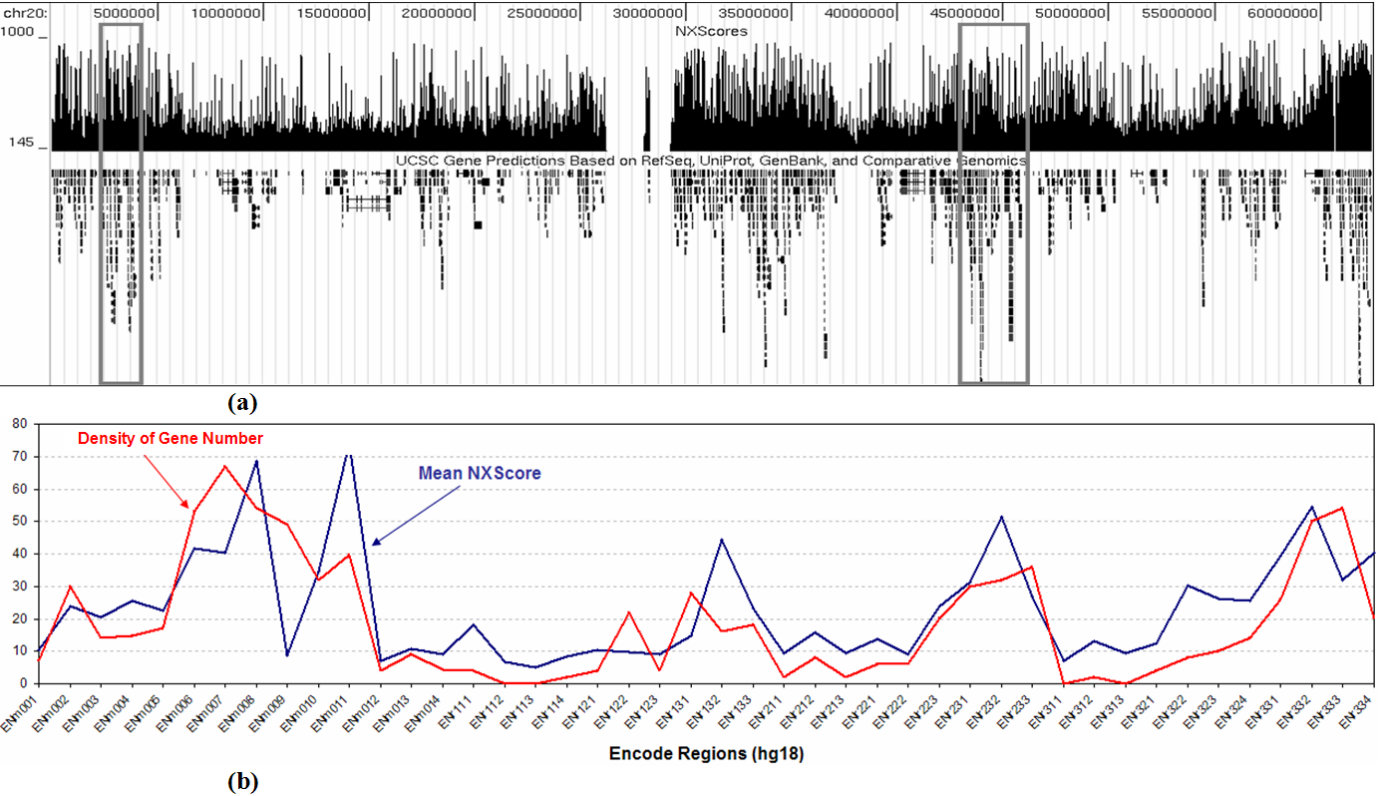
**Correlation between NXScores and gene density**. (a) Chromosome 20 is shown as an example, where the empty area in the middle is the centromere, and the boxes highlight two examples of gene-rich areas with high NXScores. The figure was prepared by uploading NXScore results as a Custom Track on the UCSC Genome Browser, and taking a snapshot with the Known Genes track. (b) The calculated correlation between NXScores and the density of gene number for the ENCODE regions of hg18.

We observed a genome-wide correlation between NXScores and gene density, such that gene-rich areas have high NXScores (Figure 2). To validate this observation, we calculated the mean NXScore for each of the ENCODE regions [22] (Human Genome, UCSC Release hg18). We then counted the number of RefSeq genes in each region, and normalized that number by the size of the corresponding ENCODE region. Figure 2b shows the mean NXScore and the density of gene number for each ENCODE region. The data sets exhibit a strong positive correlation (*r *= +0.71) based on a Pearson product-moment correlation coefficient. This confirms the observation that gene-rich areas have high NXScores. Figure 2a illustrates this trend using chromosome 20, similar figures for all the human chromosomes are available in the supplementary data files.

### Correlation with Tissue Specificity

We obtained the gene expression profiles of 19055 genes and developed a new method for ranking the tissue specificity of those genes. The available SymAtlas "tissue list" includes 79 cell types, tissues and organs, which makes it difficult to classify genes categorically into tissue specific groups. Furthermore, genes that have been classified as tissue specific by other researchers were often expressed equally in three or four different tissues. In order to overcome this problem, we refer to genes as having a wide tissue distribution if they are expressed at relatively equal levels in five tissues or more, and as having a narrow distribution if they are expressed at relatively equal levels in only one or two tissues. To follow this idea through, we needed a method of ranking genes according to their tissue distribution, so that we could correlate this with NXScores.

The RefSeq-annotated transcriptional start site (TSS) was used to identify the promoter region of each gene, and NXScores were calculated for the region TSS-1500 to TSS+500. The resulting values were used to sort the 19055 genes in ascending order (*i.e.*, from no nucleosome exclusion to complete nucleosome exclusion). The sorted list was then divided into *n *groups. The mean tissue specificity for each of these groups was calculated using a method we developed based on Grubbs' test [23], and we validated this method using an already established method for ranking tissue specificity based on Shannon entropy [24].

**Figure 3:**
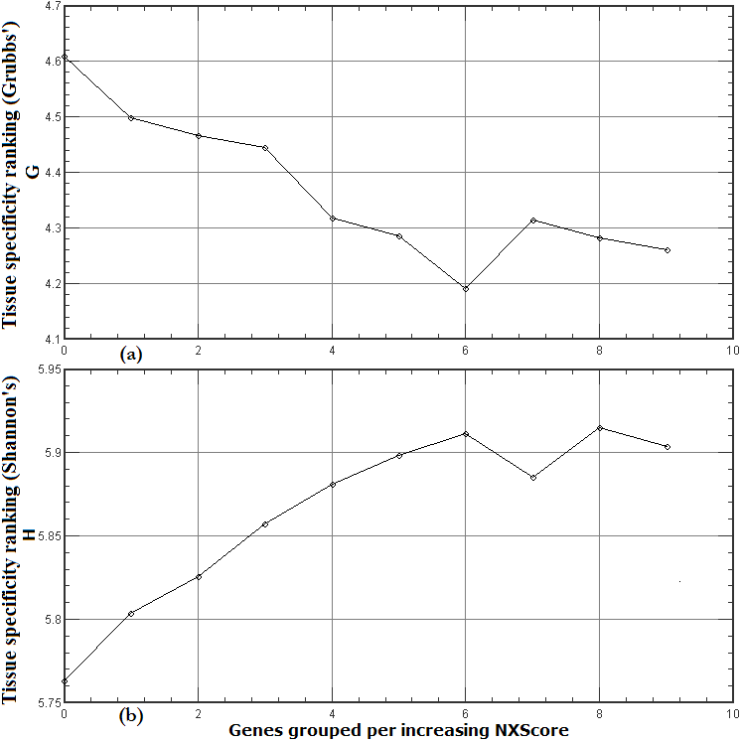
**Correlation between NXScores and tissue specificity**. The 19055 genes were arranged into 10 groups (0–9) of increasing NXScores for the -1500 to +500 promoter regions, and the mean tissue specificity level for each band was calculated using a new method based on Grubbs' test (a) and validated using a previously known method based on Shannon's entropy (b).

Grouping genes facilitated the inspection of the general trends among gene groups while filtering noise and extraneous behavior that maybe associated with specific limited number of genes (within the group). Hence, the number of groups *n *served as a zooming parameter for inspecting and visualizing such trends. Figure 3 shows the results for *n *= 10, illustrating the correlation between the tissue specificity of gene expression and NXScores. To provide a closer inspection of these trends, figures are made available in Additional file 2 for the results of groups of *n *= 5 (zoom out), *n *= 20 (zoom in), and *n *= 40 (higher zoom in).

The results show that previous localized findings [4,17] are valid on a whole genome level. There is a direct correlation between the NXScores in the promoter region and tissue specificity. The higher the NXScore of a promoter sequence, the less likely it is to include a nucleosome, and the less tissue specific the associated gene is. Given the complexity of transcriptional regulation in the eukaryotic system, there may be a few exceptions to this, but the genome-wide trend is clearly observed from our results. One could deduce from this that the transcriptional machinery has relatively unimpeded access to the TSSs of widely expressed genes. It is expected that the types of transcription factors that switch on widely expressed genes are generally not those that can tolerate the DNA being wound around a nucleosome.

**Figure 4:**
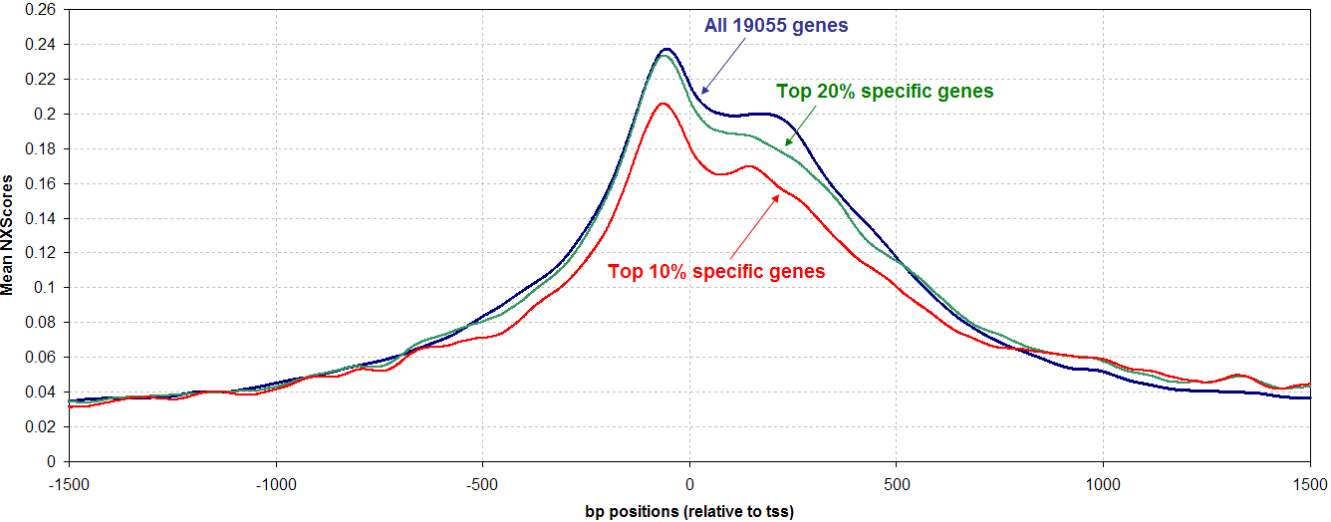
**Mean NXScores for promoter base pair positions -1500 to +1500**. The red line represents the mean NXScore across the top 10% most tissue specific genes, *i.e. *those with the narrowest tissue distribution, the green line represents the mean across the top 20%, *i.e. *a grouping of slightly wider distribution genes, and the blue line represents the mean across all the 19055 genes sampled.

To take a closer look at the promoter region, the genes were sorted according to their measure of tissue specificity, then grouped into three groups; group 1 constitutes the top 10% tissue specific genes, group 2 constitutes the top 20% tissue specific genes, while group 3 constitutes the whole collection of 19055 genes under inspection. For every gene, the NXScore for each base pair in the promoter region was calculated and then averaged separately for genes of each group. The objective was to inspect promoter NXScores profiles among genes with varying tissue specificity levels. Again, note that groups and averaging were used to inspect general trends while filtering noise and extraneous behavior that may be associated with a limited number of genes within each group. The results (Figure 4) show that the NXScore peak is approximately 30 bases upstream of the TSS, and that there is a shoulder immediately downstream from the TSS, extending approximately 250 bases into the gene. There is thus a tendency for the region surrounding the TSS to be nucleosome-free, regardless of whether the gene is widely or narrowly expressed. This presumably helps maintain the momentum of the transcriptional machinery as it moves from the TSS through the first part of the gene. After that point, there is a significant decrease in mean NXScore before it levels out, implying that the remainder of the gene is more likely occupied by nucleosomes. This is in agreement with ENCODE findings that regulatory sequences that surround transcription start sites are symmetrically distributed [25].

Our results indicate that there is a gradually increasing tendency for the promoter to be nucleosome-free the closer one gets to the TSS (Figures 1 and 4). We used the RefSeq gene-annotations of transcriptional start sites (RefSeq-TSSs), and found the average NXScore to peak about 30 bp upstream from the RefSeq-TSS. However, we also found that the RefSeq-TSSs themselves are often 20–40 bp downstream from the TSSs determined by experimental methods [5,26]. Therefore the peak of nucleosome exclusion seen in our results appears, on average, to be centered on the transcriptional start site. This is in agreement with the findings of [13], who provided experimental evidence that the region around the TSS in humans was relatively nucleosome-free.

Figure 4 highlights that all 19055 genes follow the trend explained above, and that the more tissue specific groupings follow that trend but with lower NXScore peaks. The top 10% most tissue specific genes have the lowest NXScore peak, meaning that even though their TSS region is depleted of nucleosomes, there are more exceptions to that trend in this group than there are across all the genes. This is in agreement with the previous conclusion that the more tissue-specific a gene is, the more likely it is to have nucleosomes on its promoter. The differences in NXScore peak value observed here suggest that with gradually increasing tissue specificity, nucleosome binding to promoter regions plays an increasingly important role in gene regulation.

### Correlation with Gene Expression Level

NXScores for each gene were calculated from the RefSeq-annotated TSS to the RefSeq-annotated 3'UTR end of the gene, including all exons and introns. Then the median expression level was calculated for each gene using the SymAtlas gene expression profiles [27]. We calculated the median in order to filter very high or very low expression levels that may be associated with specific tissues, since our objective for this analysis was to capture expression levels across each gene irrespective of tissue specificity. The genes were then sorted according to increasing NXScore, the sorted list was equally divided into 5 groups, and the mean expression level was calculated for each group. This grouping and the calculations undertaken were used to inspect general trends while filtering noise that may be associated with a limited number of genes within each group.

**Figure 5:**
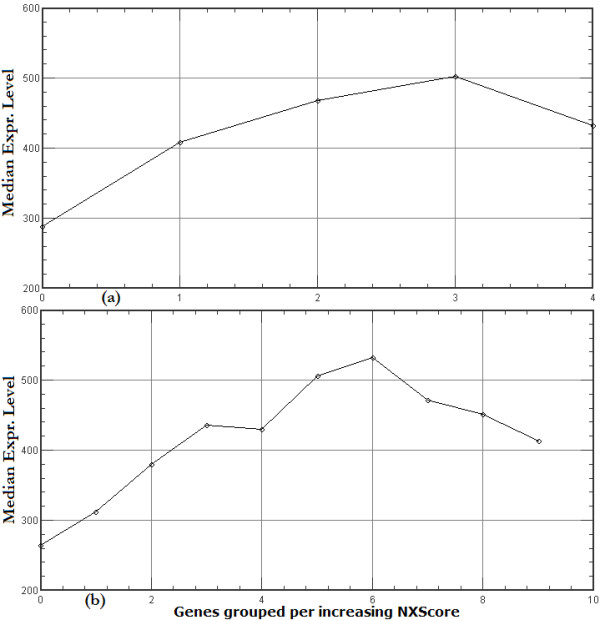
**Correlation between NXScores and gene expression levels**. The 19055 genes were first ranked according to increasing NXScore for the whole gene (TSS to end of 3'UTR), and the sorted list was divided into (a) 5 and (b) 10 groups. The mean value of the median expression level for the genes in each group was plotted. The graph shows that NXScores are positively correlated with gene expression level except at the very high NXScores, where expression levels decrease.

The data show that gene expression level is positively correlated with high NXScore (Figure 5a), and that expression level drops with very high NXScores. This can be clearly seen if we zoom in slightly and divide the data set into 10 groups, as illustrated in Figure 5b. In other words, the peak in expression level is around moderate NXScores: expression is lower when there are a lot of nucleosomes present (lower NXScore), and it is also lower when there are hardly any nucleosomes present (high NXScore). NXScores are calculated using G/C-rich sequence patterns [4], and G-C pairing involves three hydrogen bonds, whereas A-T pairing involves only two, which allows us to speculate that the lower expression levels of genes with the very high NXScores may reflect slower movement of the transcription machinery through regions of very high G-C content.

### Experimental Validation

Thus far all our observations have been *in silico*. To validate our annotations, we compared our scores to conserved nucleosome locations that have been reported in recent studies [9,13]. The study in [13] reported nucleosome occupancy on the promoter regions of several human genes, and we looked at the NXScores of those exact sequences. For further validation, we ran the NXScores algorithm on selected regions of the *Saccharomyces cereviciae *genome, namely those used in [9], to report experimentally verified nucleosome positions.

**Figure 6:**
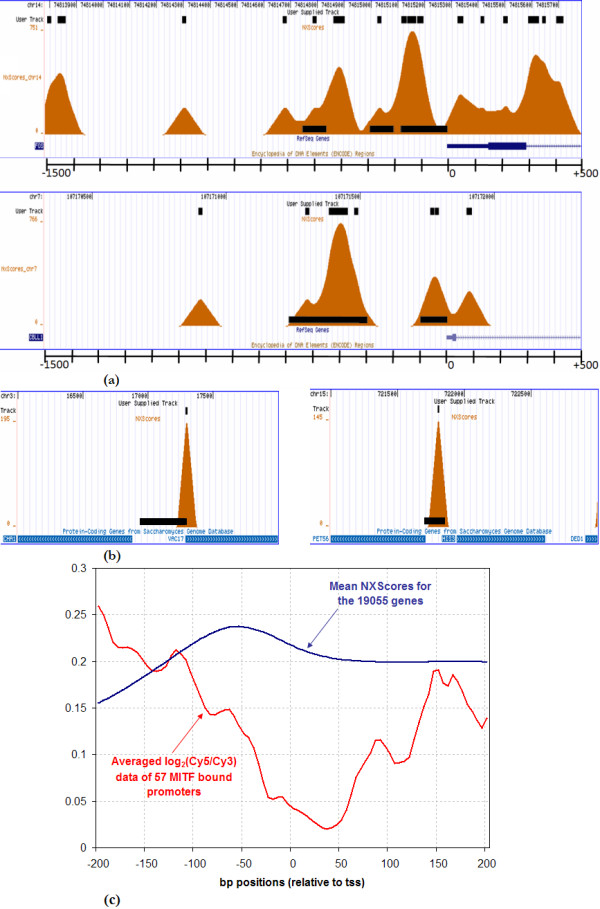
**Correlation between NXScores and experimentally verified nucleosome exclusion regions**. (a) The -1500 to +500 promoter region of the human genes FOS and CBLL1 are shown with the nucleosome positions from Ozsolak *et al. *(2007) denoted by black bars superimposed on the NXSensor graphics. The results and correlations of several other genes can be found in Additional file 3. (b) The promoter regions of the yeast benchmark genes CHA1 and HIS3 are shown with the nucleosome positions from Lee *et al. *(2007) denoted by black bars as above. The NXScore results were uploaded as a Custom Track on the UCSC Genome Browser, and a snapshot was taken with the human RefSeq genes track (a), or the protein coding genes track from yeast (b). (c) The correlation between the mean NXScores for the TSS-200 to TSS+200 promoter region of 19055 genes, and the calculated average *log*_2_(*Cy*5 = *Cy*3) data of 57 MITF-bound promoters in the human genome.

It is evident from Figure 6 (more graphs are available in Additional file 3) that although our nucleosome exclusion predictions and the experimentally verified nucleosome positions correlate well, they do not correlate exactly. In some cases, NXScores did not predict nucleosome depletion in a region where no nucleosomes were found. The results constitute a 7% false negative error margin, and for this we have two possible explanations. Firstly, we suggest that the sequences not picked up by NXScores may be regions to which nucleosomes slide according to the transcriptional activity state of the promoter at any given time. More importantly, however, these discrepancies highlight the fact that we were stringent in our choice of nucleosome exclusion sequences for our algorithm. We did not use weaker nucleosome exclusion sequences that have been reported in the literature because we wanted to have a certain level of confidence in predicting where nucleosomes will not bind, and assume that they may, at some developmental or physiological state, bind on the weaker exclusion signals [12,28].

The study in [13] calculated the average *log*_2_(*Cy*5 = *Cy*3) data of 57 MITF-bound promoters in the human genome. We compared these results with our calculated average NXScores promoter profile for the 19055 genes under inspection (Figure 6c), and obtained a medium-to-strong negative correlation (*r *= -0.47 based on Pearson product-moment correlation coefficient). This correlation is satisfactory keeping in mind that nucleosomes can slide according to the transcriptional activity of the promoter, and that our profile was calculated as a consensus promoter profile representing the 19055 genes, while the nucleosome positioning results were obtained using 57 MITF-bound promoters. In fact, overall, there were almost no examples where NXScores were high on areas that were experimentally shown to be occupied by nucleosomes.

### Correlation with DNaseI Hypersensitive sites

As a final comparison, we looked at whether nucleosome exclusion scores would correlate with DNaseI hypersensitive sites (DHSs). It is known that nucleosome-free areas are more prone to digestion by DNaseI, and it was reported in [29] that ubiquitous DHSs, shared by 6 cell lines, were found near the transcriptional start sites of some genes, implying a wide usage of that gene, or at least of that promoter. The study in [14] predicted the hydroxyl radical cleavage intensity on naked DNA for each nucleotide in the ENCODE regions. We downloaded this data for the whole set of hg18 ENCODE regions from the UCSC Genome Browser, and calculated the mean value of the predicted cleavage intensity for each region. The objective of this analysis was to investigate whether regions with high NXScores would have a high predicted cleavage intensity.

First, we calculate the NXScores for each region, and took the locations for NXScore peaks that had NXScores higher than *p*, where *p *= *μ *+ (*τ *× *s*). *μ *and *s *are the mean and standard deviation of the NXScores across the region, respectively. *τ *is a parameter for determining the height of the calculated peaks, such that the higher the *τ *value, the higher the peak value and fewer the number of the peaks across the regions, and vice versa. Next, for each peak location, we calculated the mean predicted cleavage intensity of a 147 bps neighborhood centered at the peak, and we averaged these values for all peak locations in a specific region for a specific *τ*.

In this way we were able to show that the mean predicted cleavage intensity around the peaks is higher than the mean intensity across the whole region, thus proving that regions with a high NXScore also have a high cleavage intensity.

**Figure 7:**
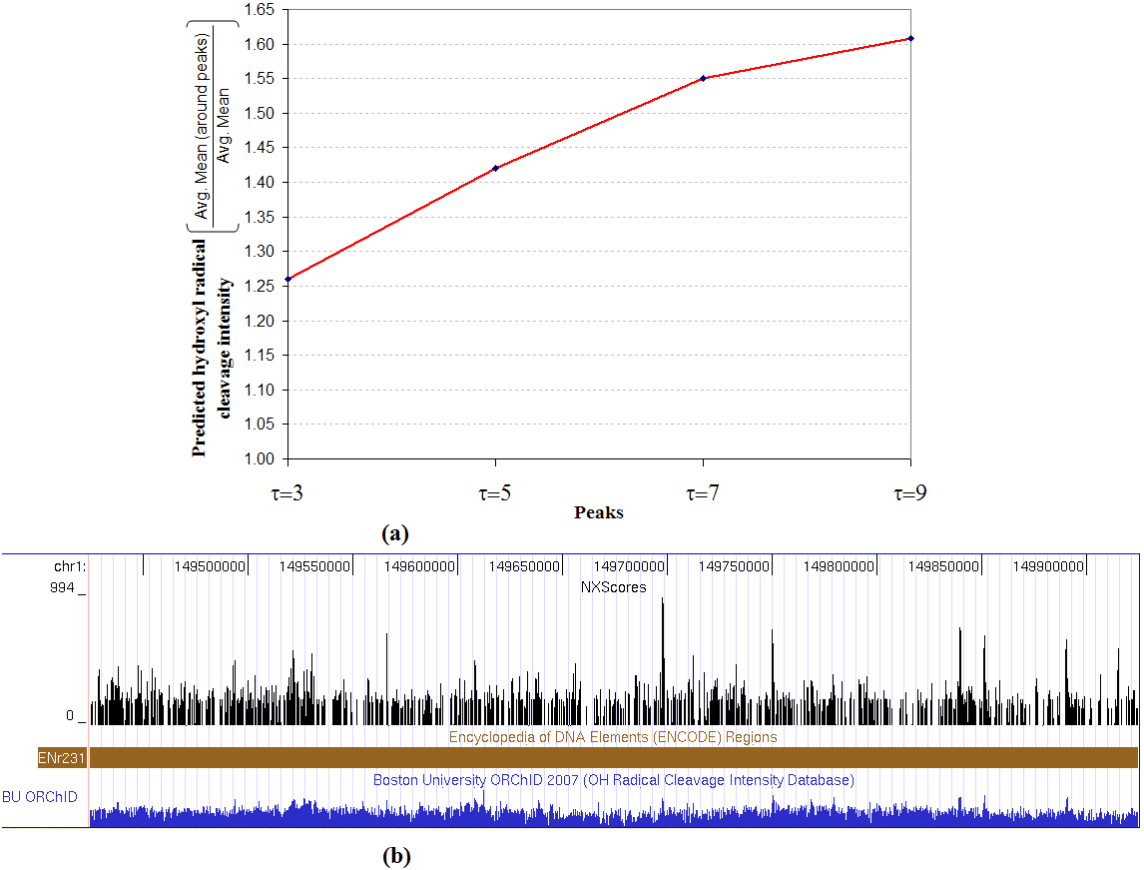
**Correlation between NXScores and DNaseI hypersensitive sites**. (a) This graph shows the ratio between the average calculated cleavage intensity around the peaks and the average cleavage intensity for that whole region, for all ENCODE regions at different *τ *values. (b) The tracks for NXScores and DNaseI hypersensitive sites are shown for ENCODE region ENr231 in a snapshot of a UCSC Genome Browser screen.

**Figure 8:**
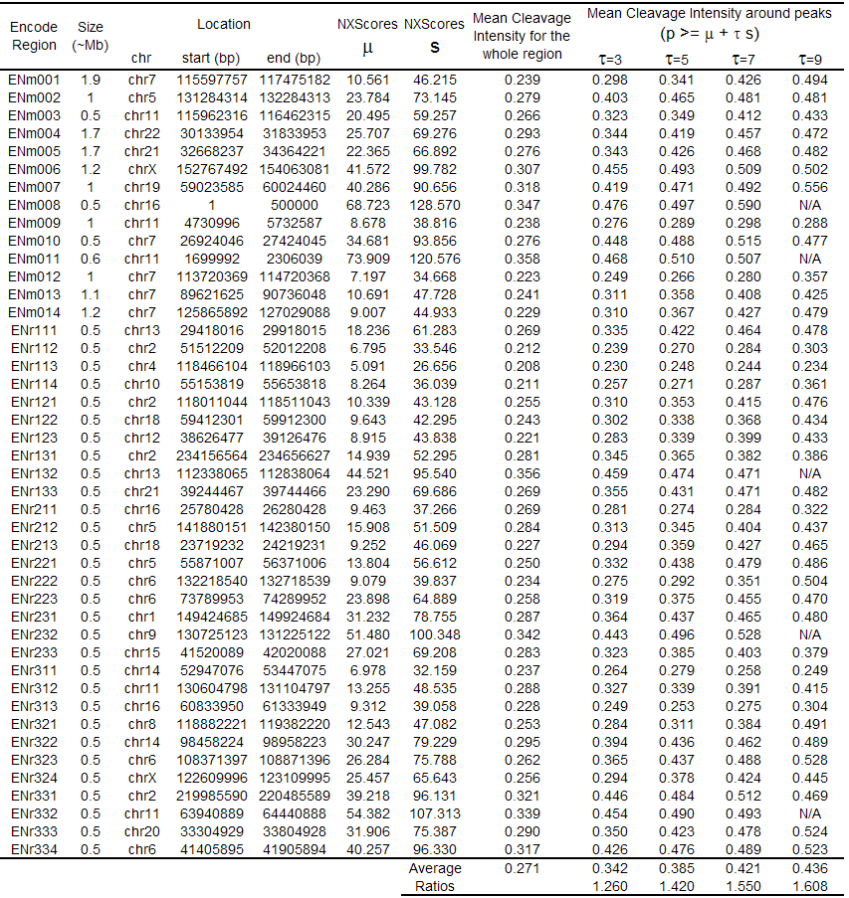
**Correlation between NXScores and DNaseI hypersensitive sites (calculations)**. This table shows the results and all intermediate calculations for the correlations between NXScores and DNaseI hypersensitive sites for all ENCODE regions of hg18.

To further investigate this, we varied *τ *from 3 to 9 and reported the results for each *τ*. As expected, the higher the *τ*, the higher the mean predicted cleavage intensity. Figure 7a shows the ratio between the average calculated cleavage intensity around the peaks and the average cleavage intensity for that whole region, for all ENCODE regions at different *τ *values. When *τ *= 3, the mean intensity increased by approximately 26%, and the intensity increased with increasing *τ*, reaching a 61% increase when *τ *= 9. Figure 7b illustrates this correlation for ENCODE region ENr231. The table in Figure 8 shows a detailed account of these calculations for each ENCODE region.

## Conclusion

A grid computing architecture was used to conduct a whole genome annotation for nucleosome exclusion regions (NXRs), and to calculate nucleosome exclusion scores (NXScores) per nucleotide across the entire human genome. The results, which are made available here, provide a measure of how likely a particular nucleotide neighborhood impairs nucleosome formation. We confirmed previous reports that nucleosomes tend to be excluded from the area surrounding the TSS of genes. We developed a new method for ranking the tissue specificity of gene use, and found that, generally speaking, the wider the tissue distribution of a gene, the fewer nucleosomes are likely to be found in the promoter region of that gene. In addition, we found that high NXScores were correlated with moderate gene expression levels, and with the presence of DNaseI hypersensitive sites.

There is increasing evidence in the literature that chromatin structure plays a crucial role in gene regulation [14,29]. These results contribute to our understanding of the relationships between nucleosome distribution and gene regulation.

## Methods

### Grid Computing Architecture

This study used a pilot grid computing architecture for computationally intensive DNA sequence analysis. The grid fabric is part of the IBM sponsored LAGrid [30] project. The architecture is composed of a group of *utility *and *computational *services. Utility services facilitate the automated composition of workflows, capture domain semantics, and address the distributed nature of the computations. The computational services are web services wrappers for sequence manipulation tools and algorithms, such as pattern searching and clustering. This architecture was designed with the objective of applying real-time, high performance capabilities to computationally intensive sequence analysis questions. *In silico *annotations of nucleosome exclusion regions across the whole human genome offered an opportunity to test and validate this design. A detailed description of the architecture and services was reported in [31].

### Locating Nucleosome Exclusion Regions

We used a slightly modified version of nucleosome exclusion patterns identified in [4], which in turn were based on experimental data from a variety of sources [32-34]. These sequences were used to locate nucleosome exclusion regions (NXRs) throughout the human genome:

[(*G*/*C*)_3_*N*_2_]_≥3_; e.g.: GGCAACGCTTGGGTA

*A*_≥10_(= *T*_≥10_); e.g.: AAAAAAAAAA, TTTTTTTTTT

It should be noted that our algorithm did not include sequences that had a weaker tendency to exclude nucleosomes, or that were rare on a genome-wide level, such as TGGA repeats [35]. This is because nucleosomes are known to slide [15] and we did not want to annotate a weak signal in case nucleosomes could slide into that particular region. Having said that, we do intend to update the annotations on the supporting online website when other strong exclusion sequences are reported and verified.

The hg18 (March 2006) human genome build was downloaded from the UCSC Genome Browser, and scanned base by base for NXRs. NXRs were annotated, and overlapping patterns were merged into one contiguous region in the final annotation. The annotations were compiled into a well supported exchange format for Feature description, GFF.

### Nucleosome Exclusion Score Calculation

The nucleosome exclusion score (NXScore) measures the tendency of a specific DNA region to exclude nucleosomes. In order to have a continuous score across query sequences of variable length, the NXScore for each single base pair was calculated relative to a 147 base pair window, defined as the *neighborhood *of a particular nucleotide, centered at that nucleotide. The results per nucleotide are used to calculate the NXScore for any given region, as shown below:

#### • NXScore calculation for a single base pair

Calculating the NXScore per nucleotide depends on the density of NXRs in the 147 bps neighborhood of that nucleotide, however to fine tune our score calculation we specifically evaluated the weighted density of NXRs in the neighborhood. The idea behind the weighted density is to assign higher weights to NXRs close to the base pair under calculation than distant NXRs. We used a simple linearly decreasing weighting function, after finding that other functions yielded similar results, and maintaining that our main concern was identifying the peaks rather than the rate of change of the scores. For example, the score for a nucleotide whose 147 bp neighborhood contains one NXR of length *x *and located at either end of the neighborhood should be less than the score for a nucleotide whose 147 bp window contains one NXR of the same length *x *but at the center of the window (*i.e.*, surrounding the nucleotide in question).

NXScores can take the values 0 to 1 inclusive, such that if a nucleotide is centered in a neighborhood that is full of NXRs, then its NXScore will be equal to one. On the other hand, if the neighborhood is free from NXRs then the NXScore for that nucleotide will be zero. Figure 8 illustrates an example of NXRs and NXScores of a particular gene, chosen from chromosome 21. For display purposes, the NXScores in this figure and the rest of the figures in the paper were scaled up to span the range from 0 to 1000 inclusive.

#### • NXScore calculation for a sequence

Having defined the NXScores for single nucleotides, and given a DNA sequence of length *n *bp, its NXScore *S*_*n *_is defined as the average NXScores for the *n *bp that make up that sequence. This can be represented by the formula: $S_{n}=\frac{1}{n}\sum_{i=1}^{n}s_{i}$, where *s*_*i *_is the NXScore for bp *i*, and the summation is over the *n *bp.

NXScores annotations for the whole human genome are available in wiggle format [20] from Additional file 6.

### Tissue Specificity Measures

A number of methods, based on microarray gene expression datasets, have been proposed for measuring the tissue specificity of gene use. Despite the inherent limitations of comparing microarray datasets, some methods have been able to describe trends in tissue specificity. In [18], the effectiveness of using Shannon entropy was demonstrated for ranking genes according to their tissue specificity, from narrow or tissue-specific expression, to wide or ubiquitous expression. Shannon entropy was used and updated in [36]. Earlier, a method derived from Akaike's information criterion, which was originally developed to detect outliers in a data set, was applied in [37], and was used to rank genes according to their tissue specificity. Using the GNF-SymAtlas [38] gene expression dataset [27], we categorized known genes according to their tissue specificity levels, and investigated their possible correlation to NXScores.

#### Data Preparation

The GNF-SymAtlas dataset contains 44,775 expression profiles across 79 human tissues and cell types and is itemized by oligonucleotide probes [27]. First, all non-specific and partially-specific microarray probe sets were removed from our dataset, leaving only the specific target data, with each probe corresponding typically to only one gene. This probe set was then joined with the Known Gene database of the UCSC Genome Browser [39], from which information on the chromosomal location of the gene, and its transcriptional start and end positions was extracted. Any further redundancies were filtered out at this step, resulting in 19055 genes. The promoter region sequence (-1500 to +500) for each of these genes was downloaded and fed for analysis through processing and pattern matching modules. A distributed grid computing architecture was used, in which the modules were wrapped as web services and distributed among a number of grid nodes. The development was done using Java, and some modules utilized BioJava APIs [40].

#### Algorithms Used

We propose a new and efficient technique for ranking genes according to their tissue specificity, based on Grubbs' outliers test [23]. To validate our results we also used the previously published ranking mechanism utilizing Shannon's entropy. Both techniques gave almost the same results, verifying that this use of Grubbs' test is valid. It should be noted that the proposed technique in this study has the advantage of being able to detect both up-regulated and down-regulated genes in a microarray data set. We defined up-regulated genes as those that are expressed at a significantly high level in a limited group of tissues compared to their expression in other tissues, and down-regulated genes as those that are expressed at significantly lower levels in a limited group of tissues compared to mid- to high-expression in other tissues. Even though for this particular study we only used up-regulated genes, the applicability of this method is valid for other data sets.

**Figure 9:**
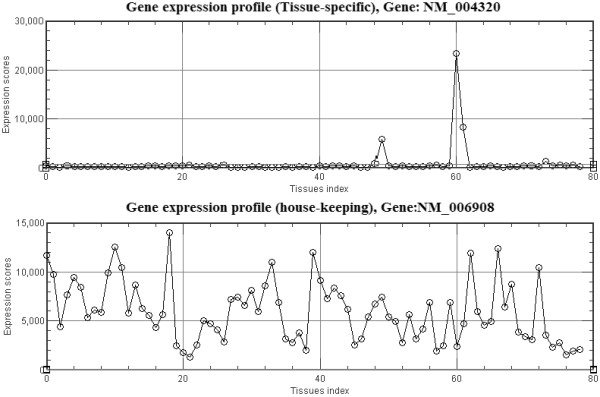
**Tissue-specific and wide-use genes**. Examples of the expression profile for a) a tissue-specific gene, NM_004320, and b) a wide-use gene, NM_152422.

Figure 9 shows examples of the expression profiles of a tissue-specific gene (NM_004320, ATP2A1) and a gene that has a wide tissue distribution (NM_006908, RAC1). The *x – axis *represents the tissues index, while the *y – axis *depicts the expression scores. Strictly speaking, microarray data are not quantitative measures of expression levels, but they do give some indication of the trends. These values were used in the tissue specificity ranking calculations, detailed as follows:

• Grubbs' outliers test:

The Grubbs' test [23], also known as the maximum normalized residual test, can be used to test for outliers in a univariate data set. Given the expression profile of a gene, the Grubbs' test *G *can be calculated as $G=\frac{max(w_{t}-\bar{w})}{std}$, where, $std=\sqrt{\frac{1}{n-1}\sum_{t=1}^{n}{\left(w_{t}-\bar{w}\right)}^{2}},\bar{w}=\frac{\sum_{t=1}^{n}w_{t}}{n}$, *w*_*t *_is the expression score for tissue *t*, *std *is the standard deviation for the expression profile, and $\bar{w}$ is the mean expression score. The more specific the gene, the higher the *G *value, and vice versa. While this formula for G identifies up-regulated genes, replacing *max *with *min *can identify down-regulated genes.

• Shannon's entropy:

The concept of Shannon's entropy [24] has a central role in information theory, and is sometimes referred to as the measure of uncertainty. The entropy of a random variable is defined in terms of its probability distribution, and has been shown to be a good measure of randomness or uncertainty. The entropy is maximum when the variable is uniformly distributed, *i.e.*, it exhibits the highest uncertainty.

Given a gene expression profile similar to those in Figure 8, the Shannon entropy (*H*) can be calculated as $H=\sum_{t=1}^{n}p_{t}.log_{2}(p_{t})$, and, $p_{t}=\frac{w_{t}}{\sum_{t=1}^{n}w_{t}}$, where *w*_*t *_represents the expression score for tissue *t*, and *p*_*t *_is calculated by normalizing this value relative to the sum of expression scores for all tissues. The more specific the gene, the less its entropy, and vice versa.

## Competing interests

The authors declare that they have no competing interests.

## Authors' contributions

AR conducted the computational analysis, implemented the algorithm, and drafted the initial manuscript; AY helped with the computational design and algorithm development; PL conceived the original idea and contributed to discussions throughout; SK lead the project, helped with biological interpretation of the results, and completed the manuscript. All authors read and approved the final manuscript.

## Supplementary Material

Additional file 1 NXScore profiles for all 23 chromosomes and the mitochondrial chromosome. Click here for file

Additional file 2 Graphs of additional groupings for Figure 3. Click here for file

Additional file 3 Graphs of additional genes for Figure 6. Click here for file

Additional file 4 Genes used and their tissue specificity. An SQL file containing the 19055 genes used in this study and their tissue specificity level according to Shannon's entropy. Click here for file

Additional file 5 Nucleosome exclusion regions in GFF format. A zip file containing the whole annotations for human genome nucleosome exclusion regions in gff format, a single *.gff file for each chromosome. Click here for file

Additional file 6 Nucleosome exclusion scores in wiggle format. A zip file containing the whole annotations for human genome nucleosome exclusion scores in wiggle format, a single *.wig file for each chromosome. Click here for file
