# Supplementary figures and images for: Prediction and analysis of nucleosome exclusion regions in the human genome
Source: BMC Genomics. 2008 Apr 22;9:186. doi: 10.1186/1471-2164-9-186 (PMC2386137; doi:10.1186/1471-2164-9-186)

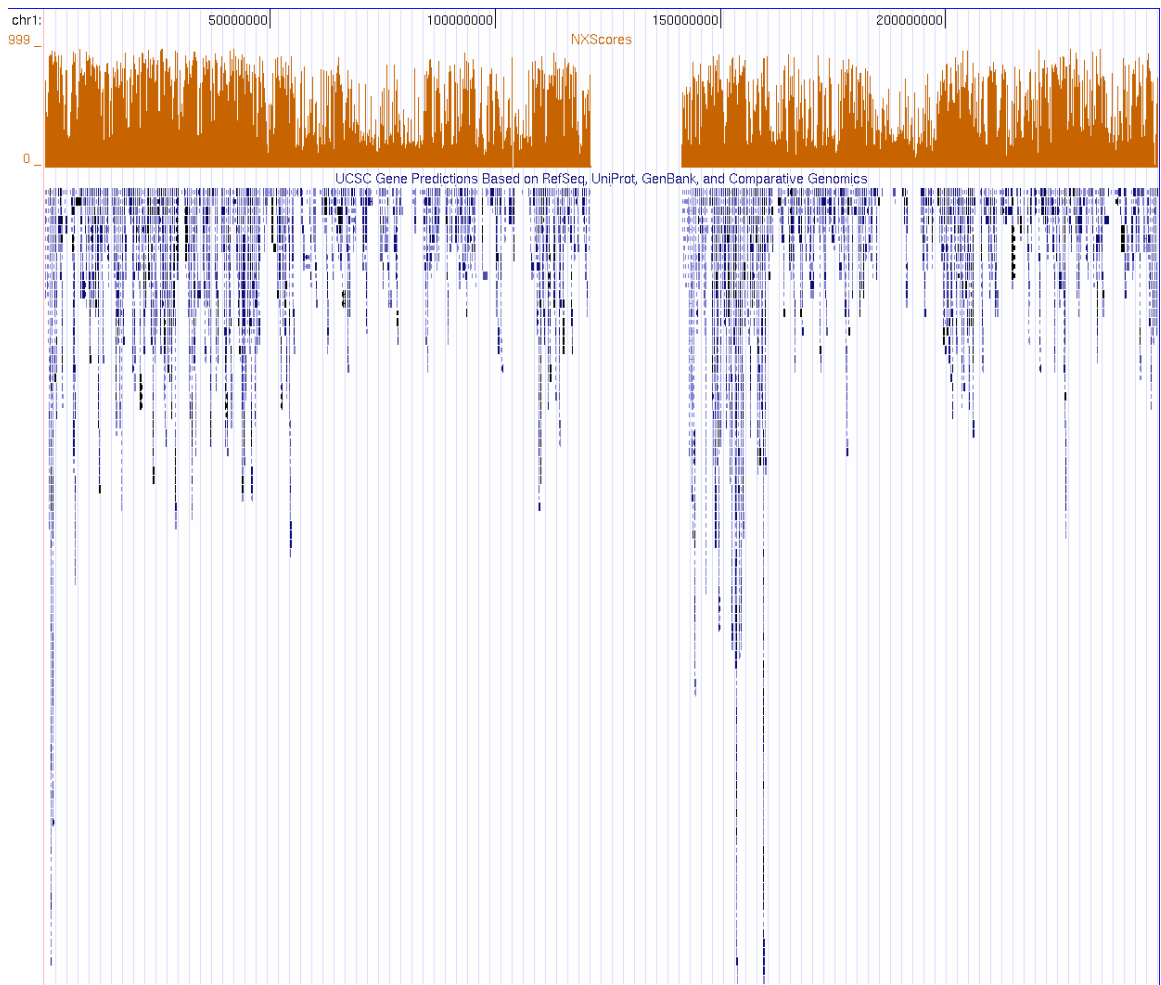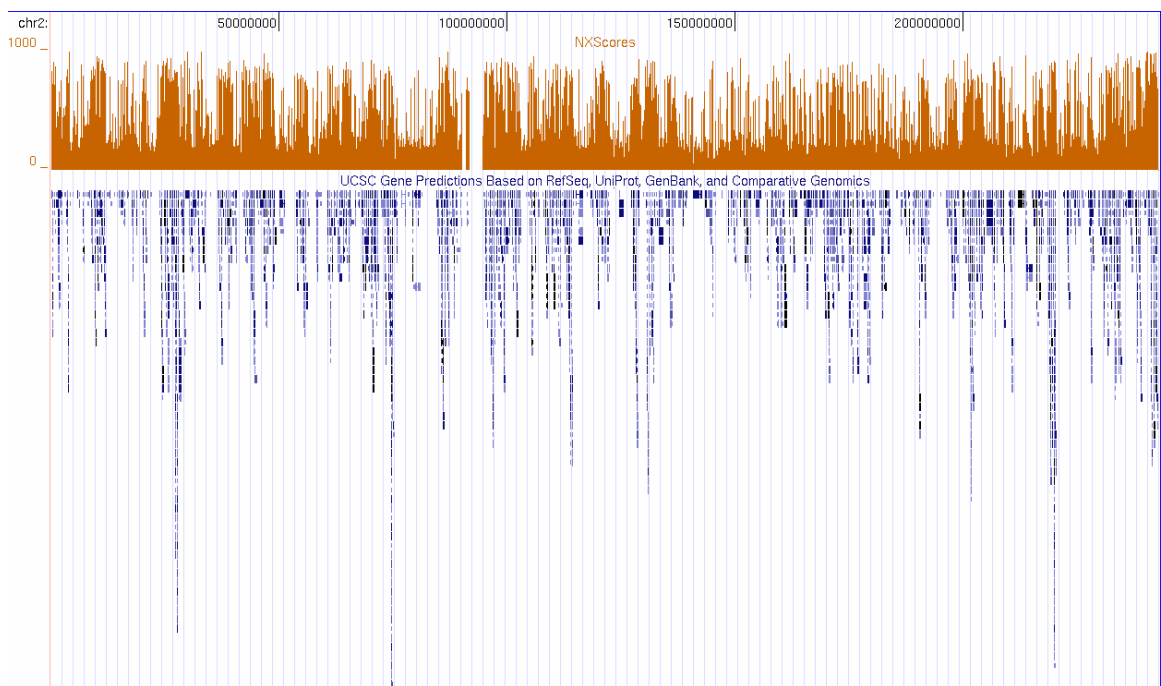

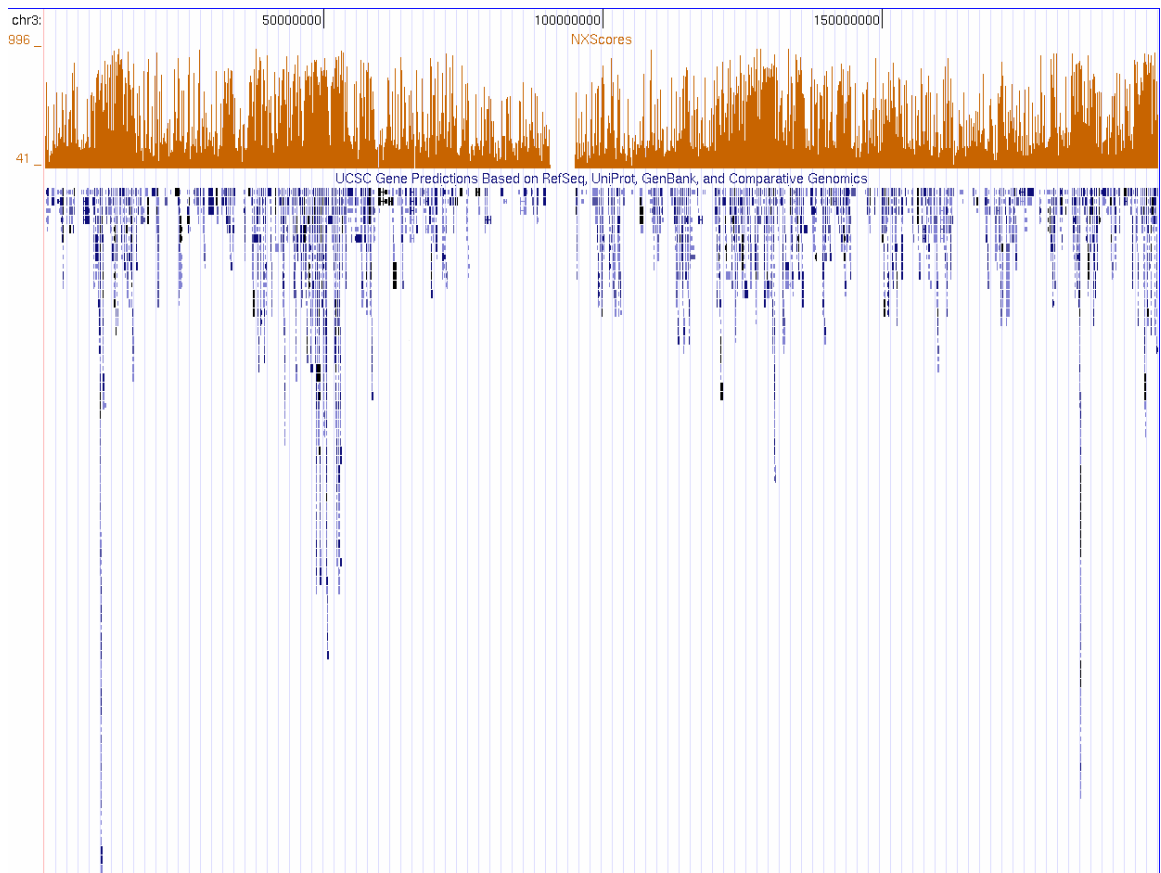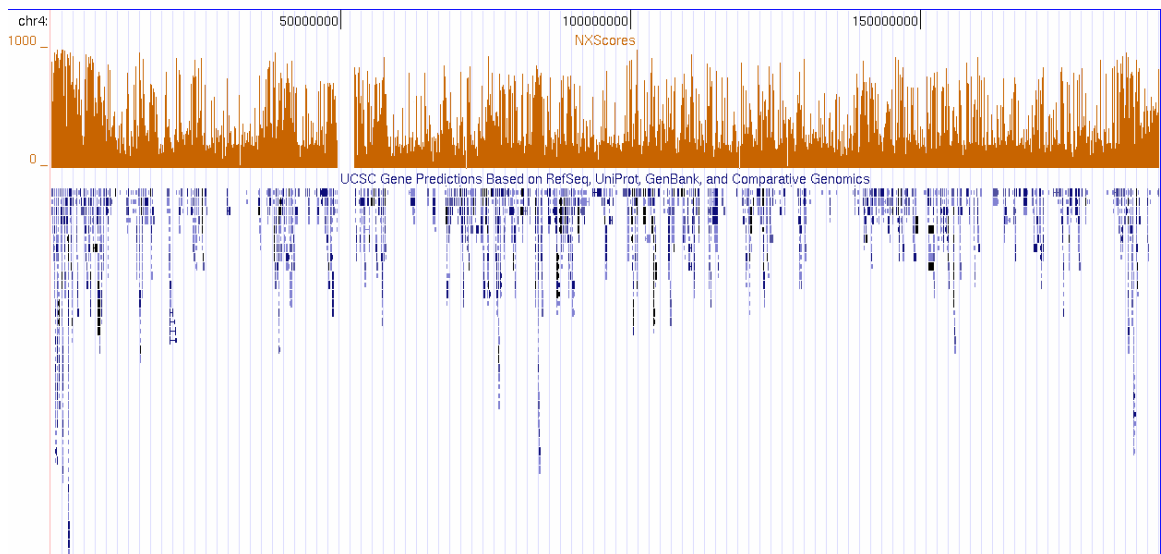

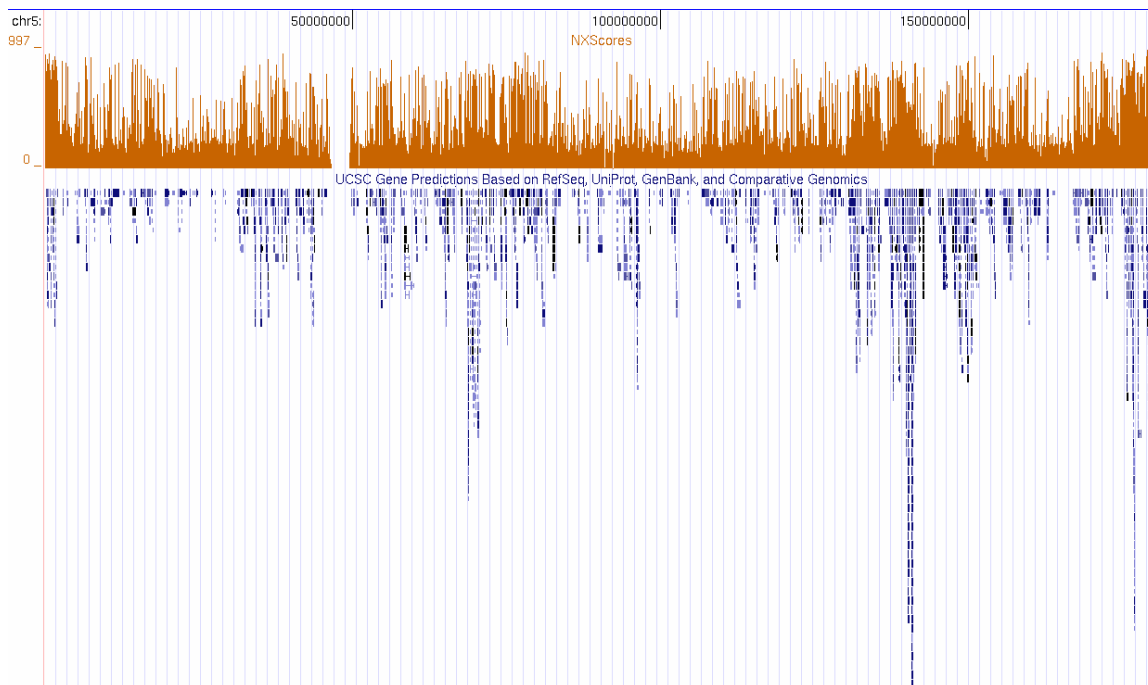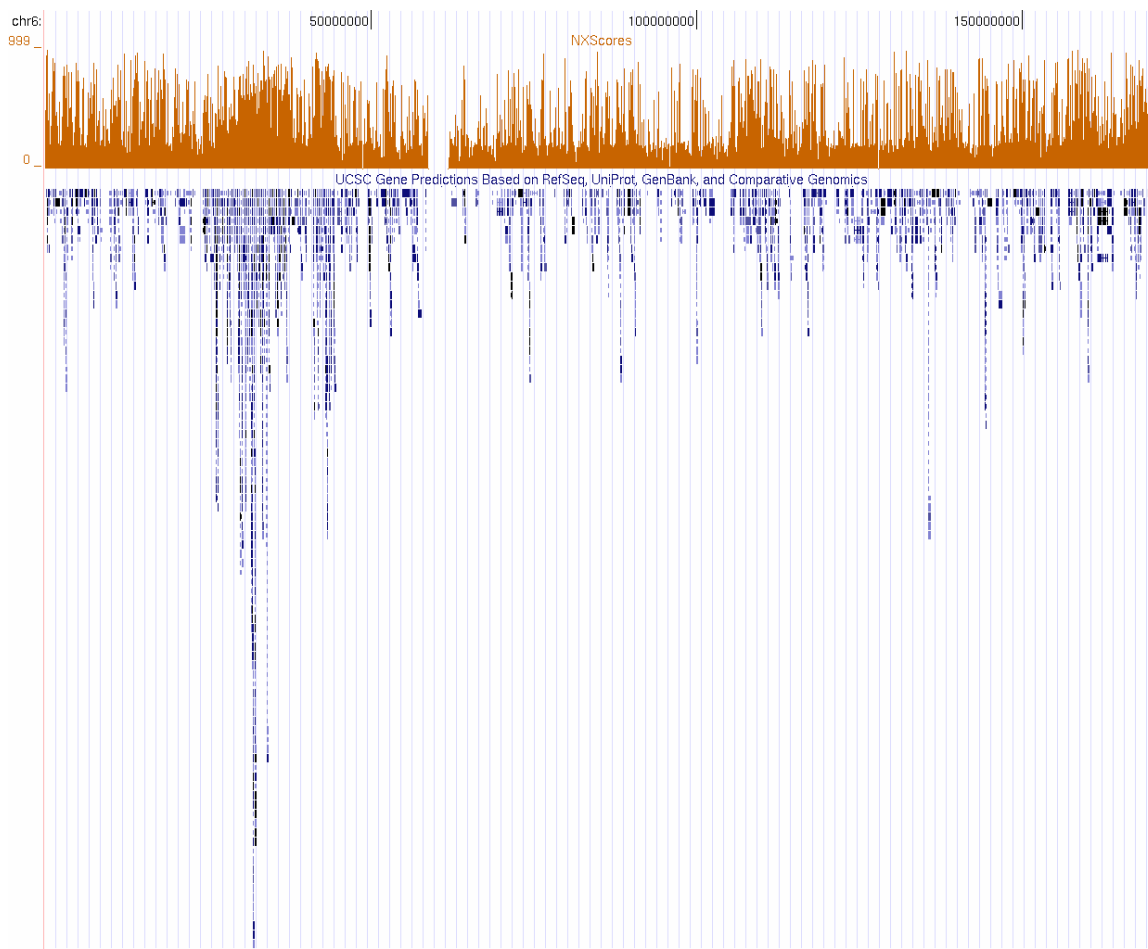

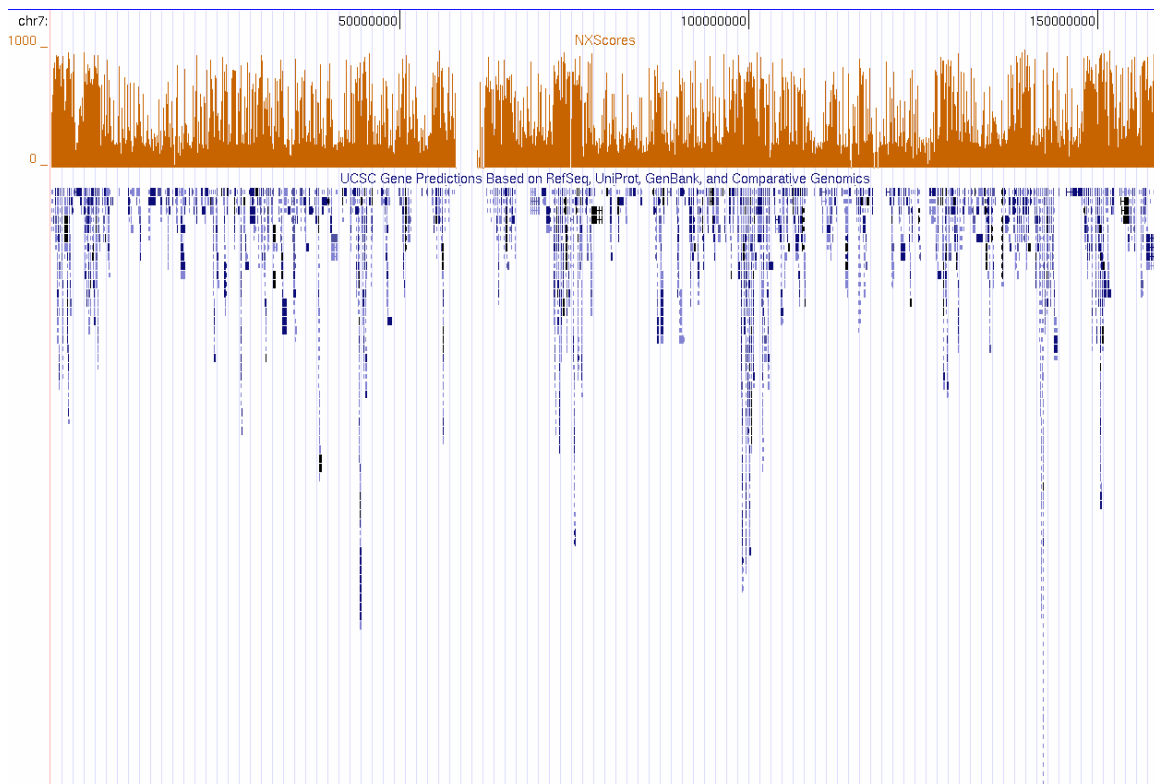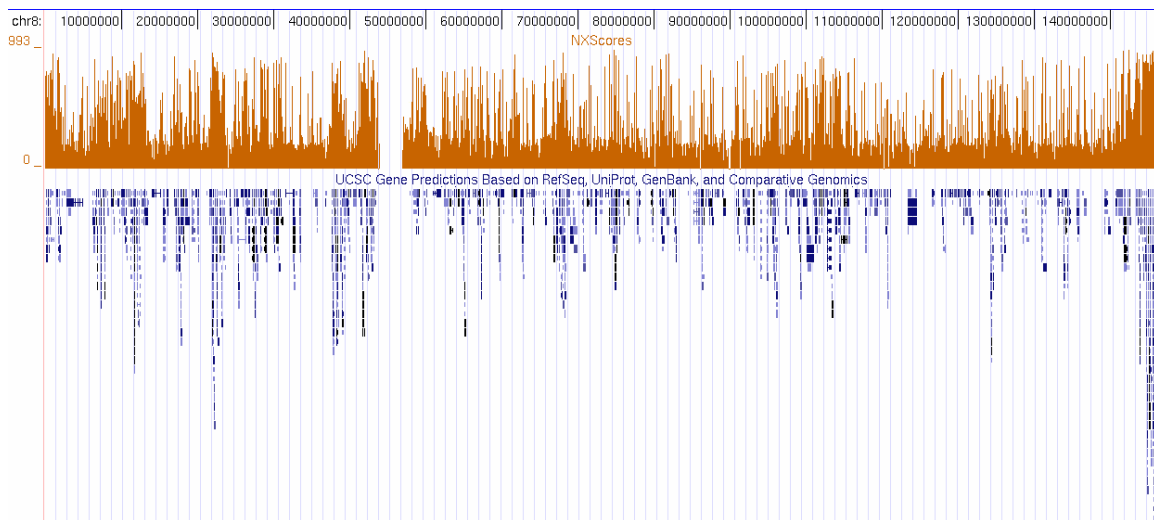

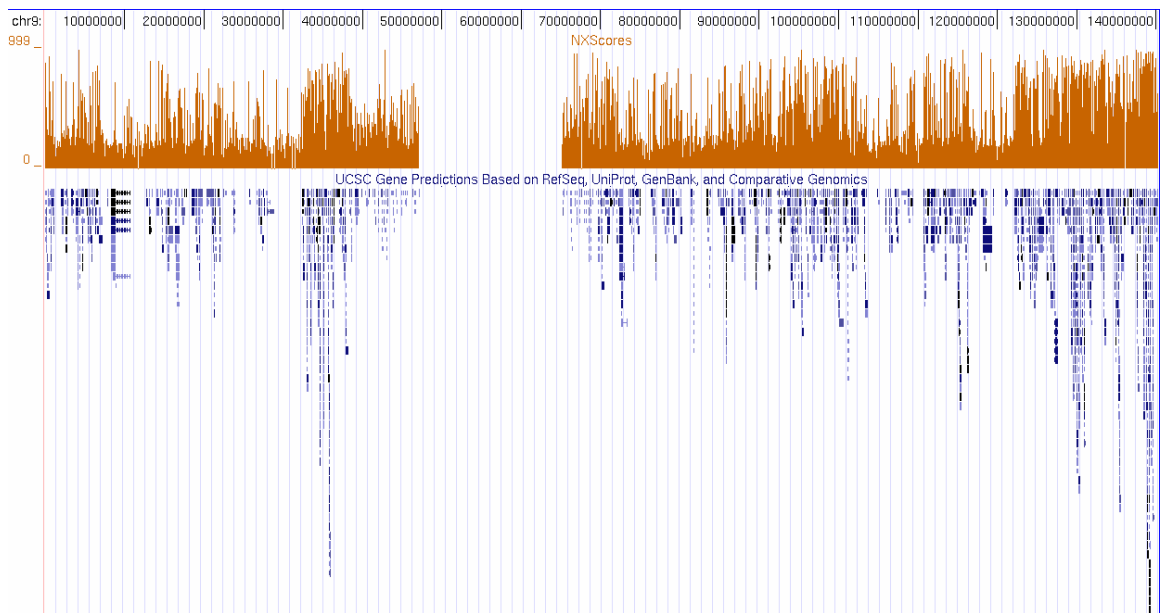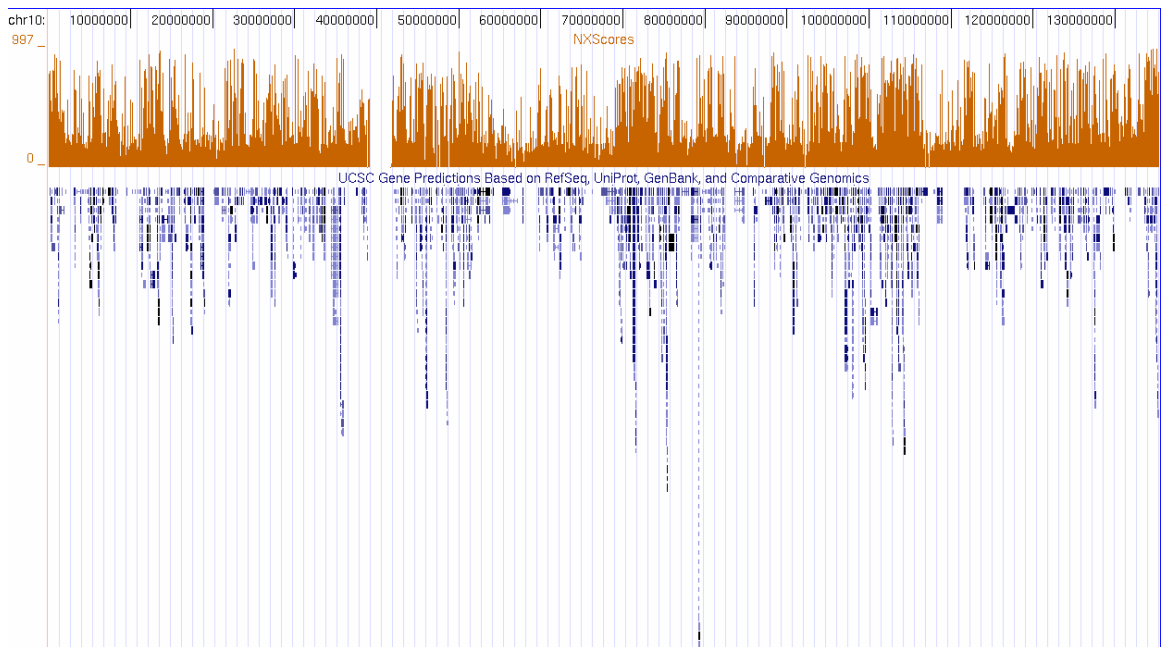

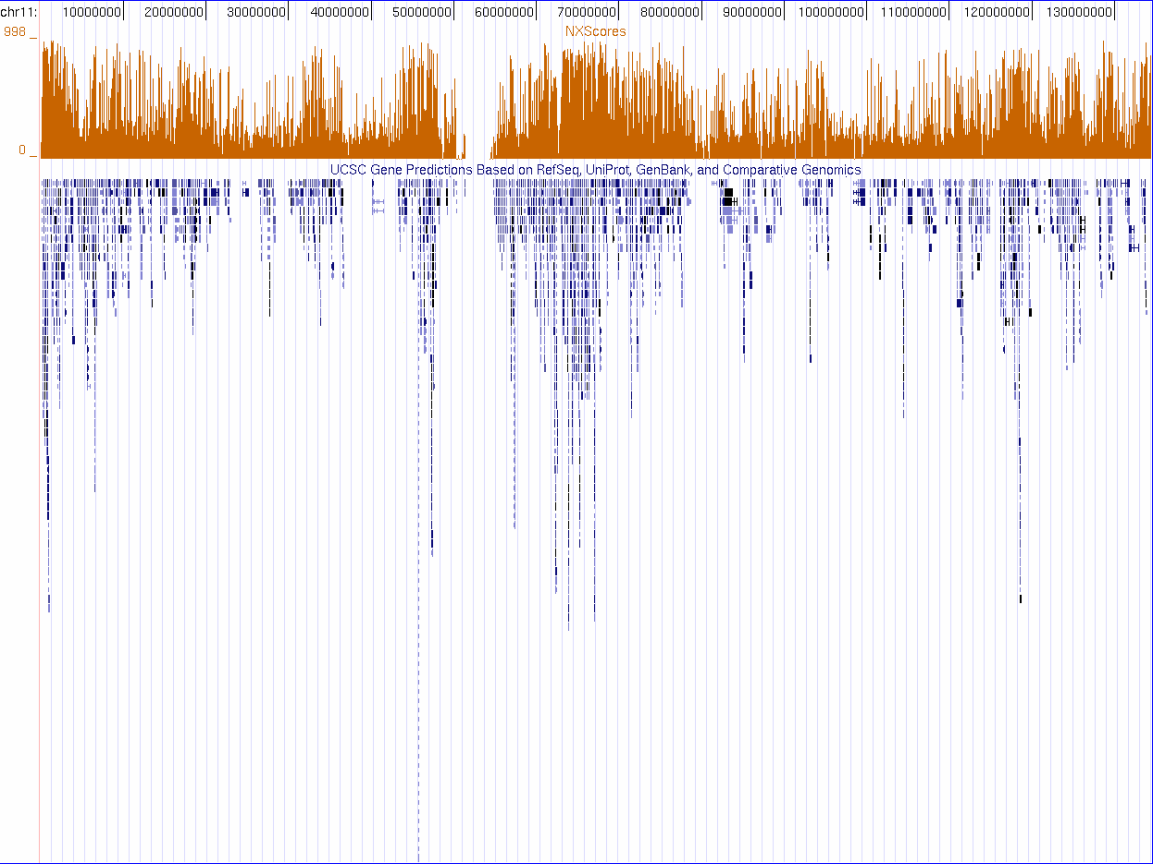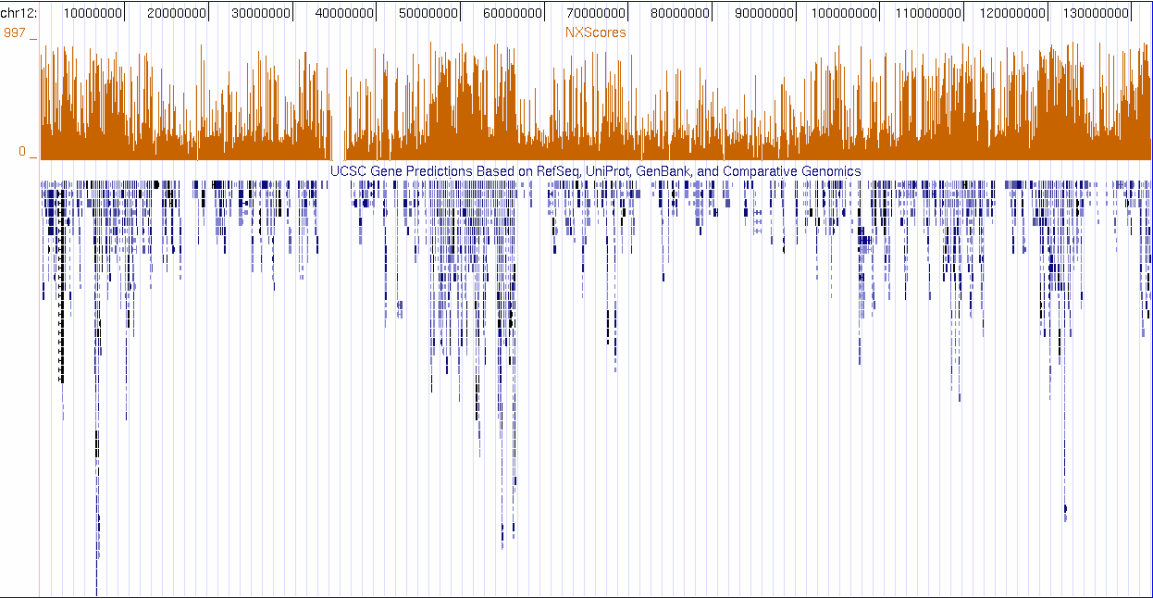

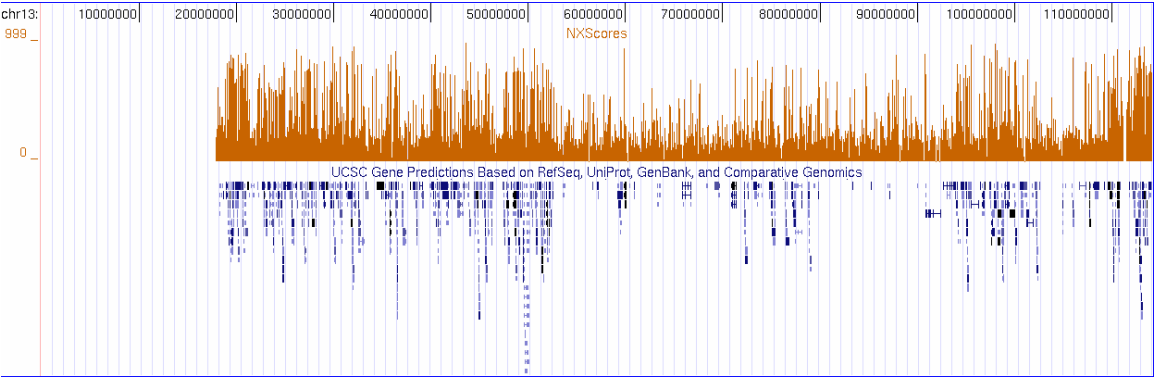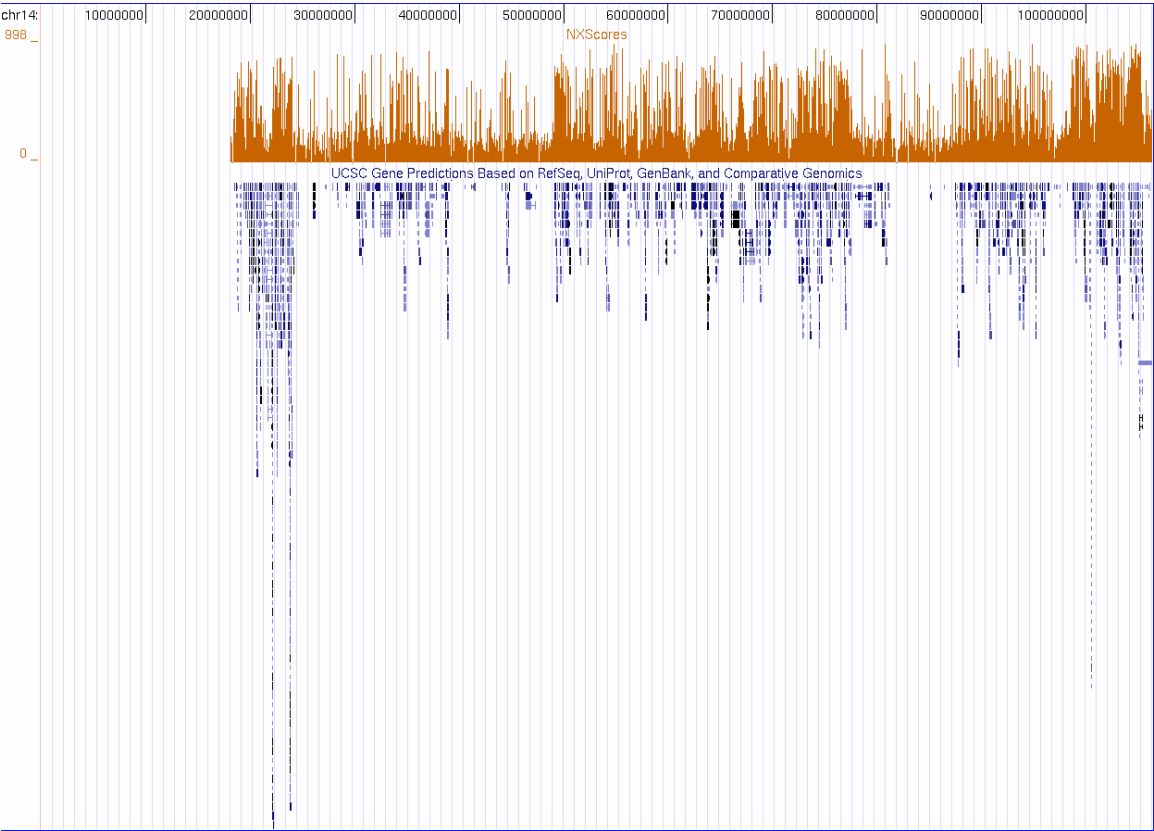

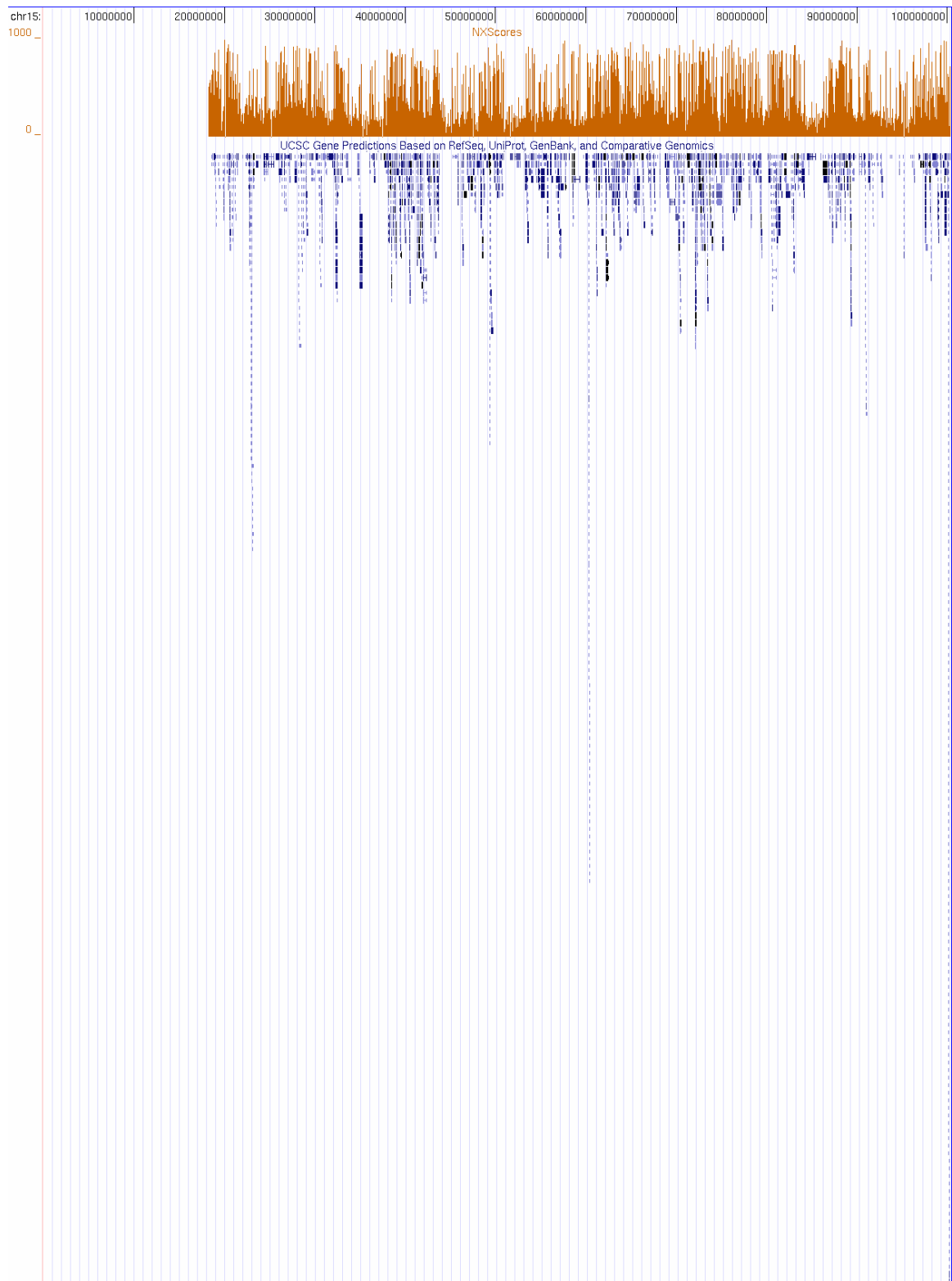

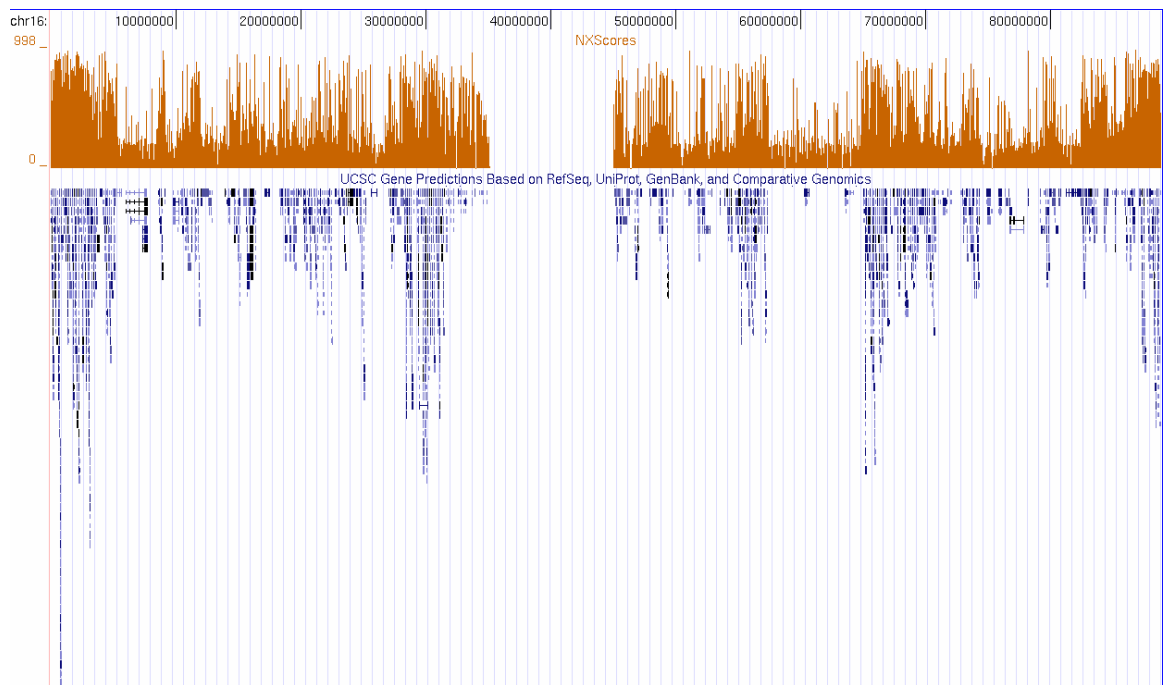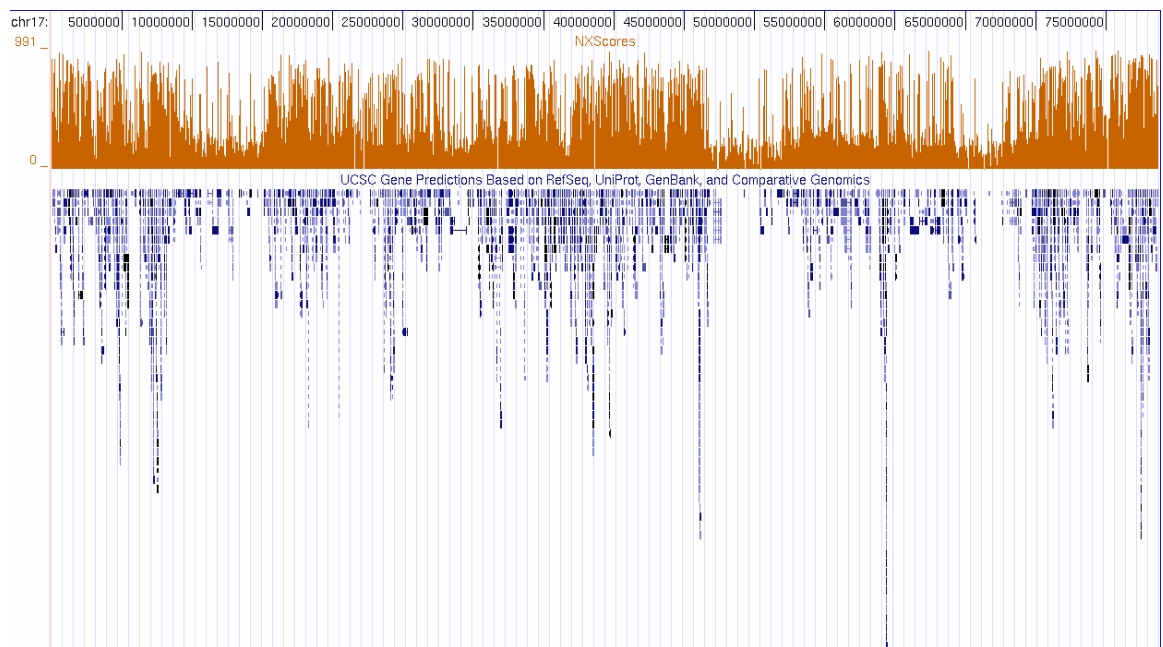

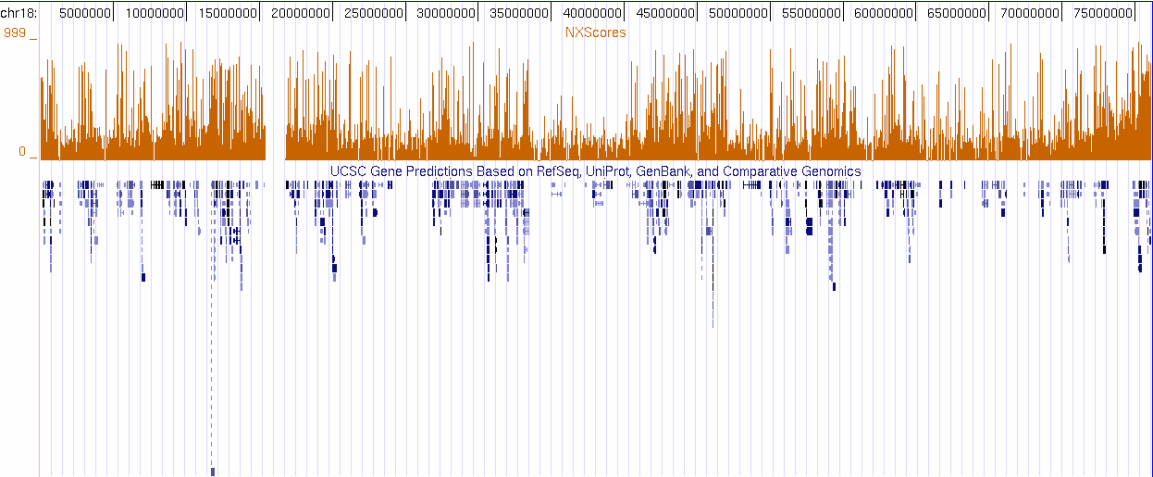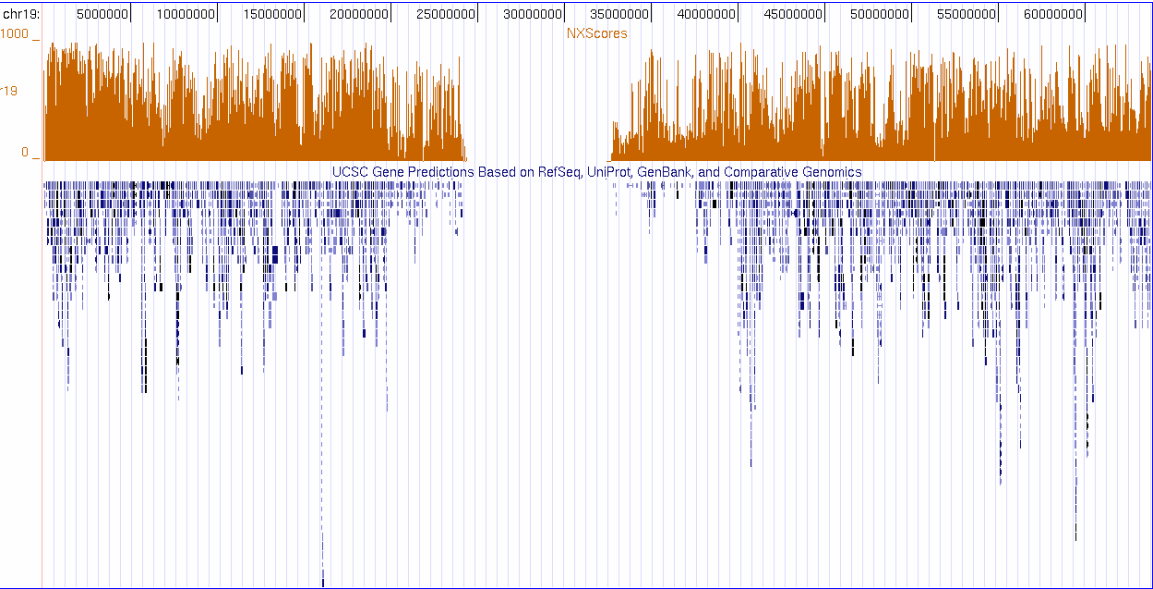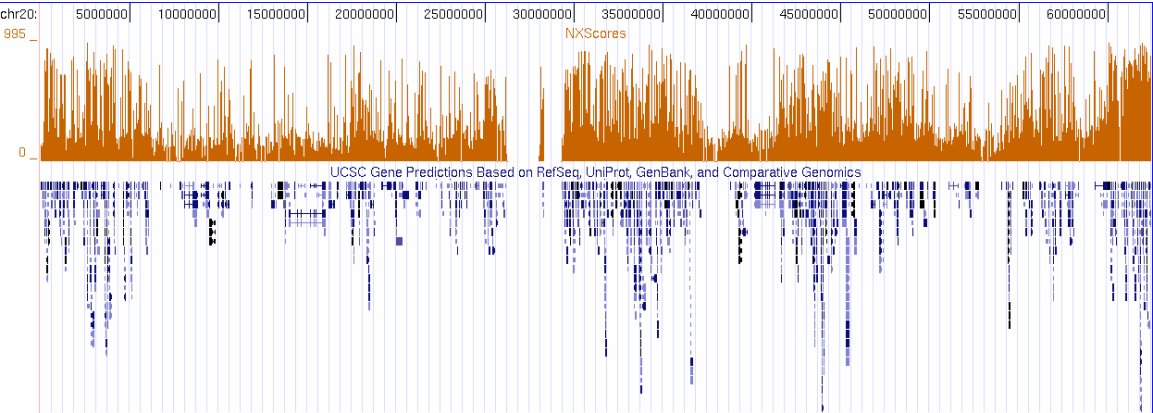

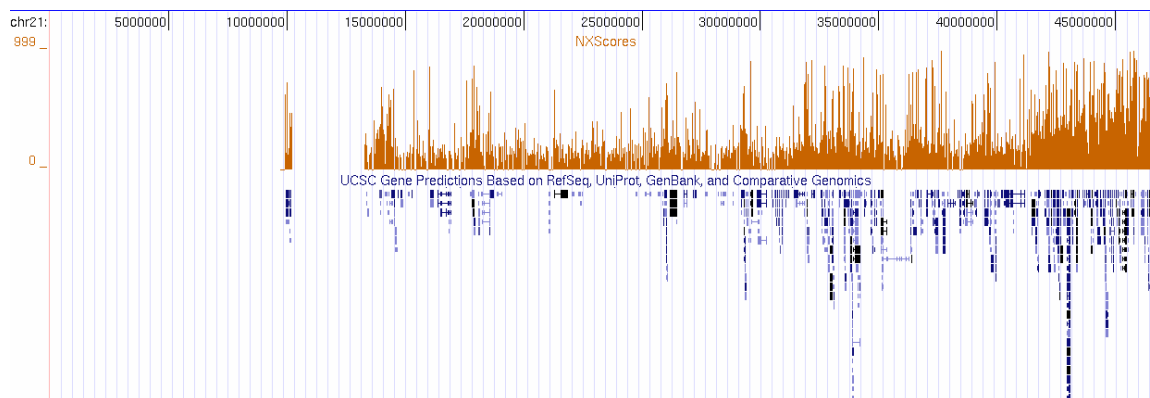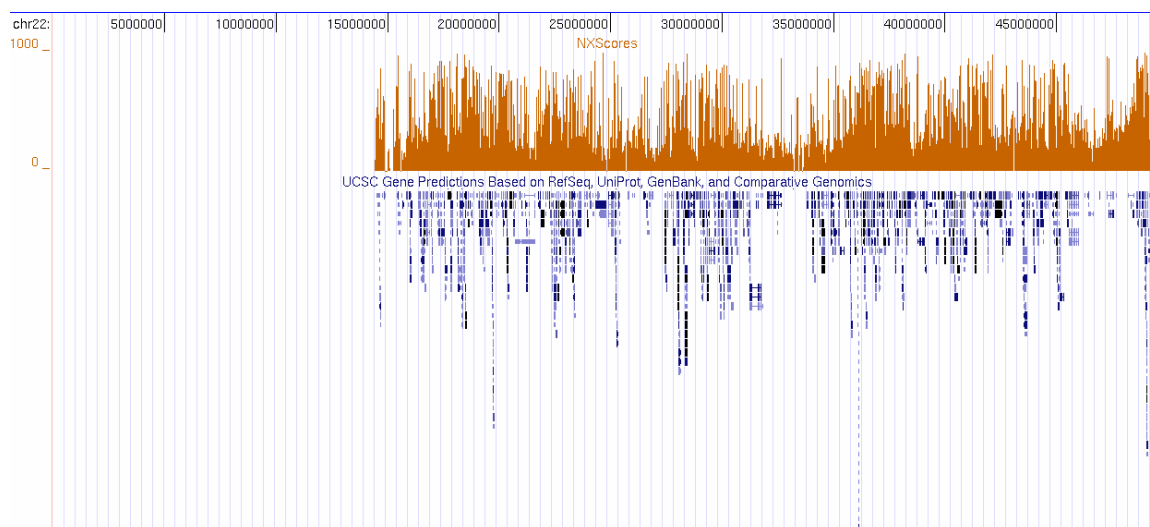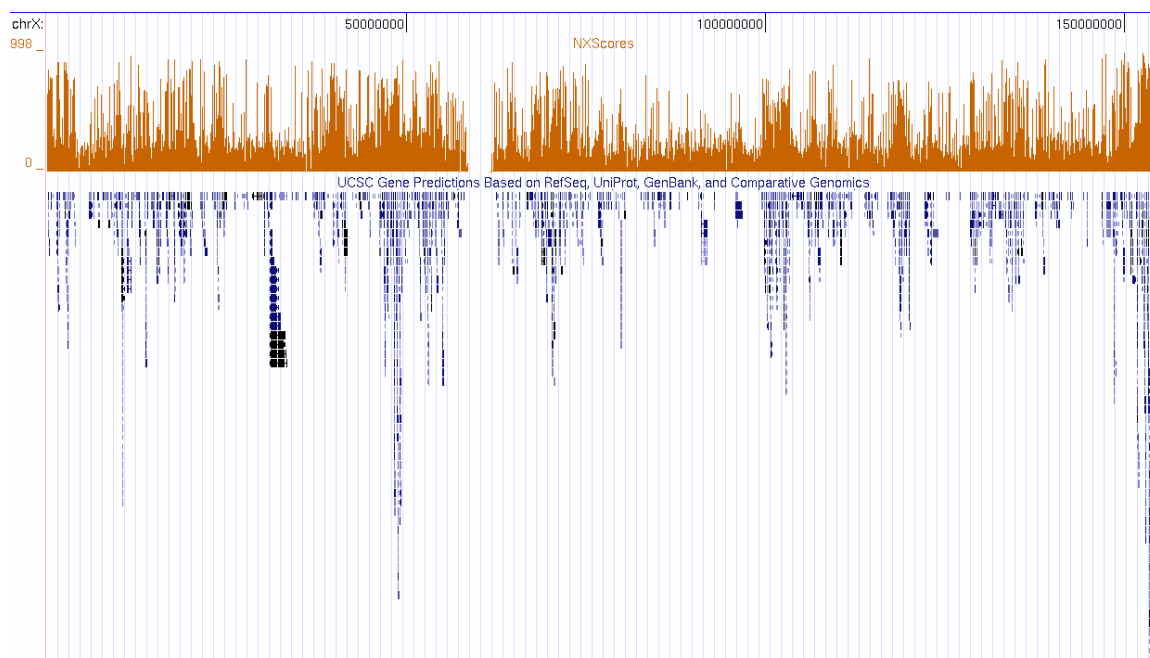

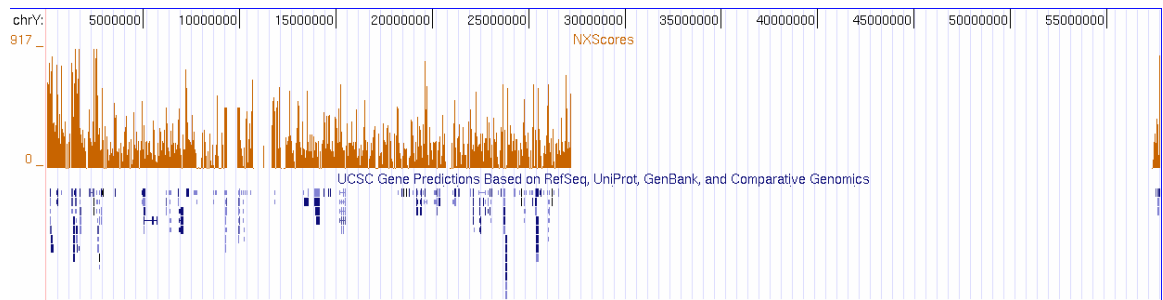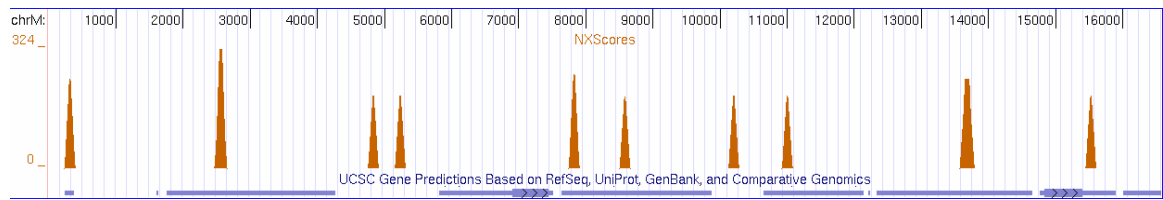

Supplement: Additional file 1 — NXScore profiles for all 23 chromosomes and the mitochondrial chromosome. [file 1471-2164-9-186-S1.pdf]
