# Supplementary material for: Prediction and analysis of nucleosome exclusion regions in the human genome
Source: BMC Genomics. 2008 Apr 22;9:186. doi: 10.1186/1471-2164-9-186 (PMC2386137; doi:10.1186/1471-2164-9-186)
Supplement: Additional file 2 — Graphs of additional groupings for Figure 3. [file 1471-2164-9-186-S2.pdf]

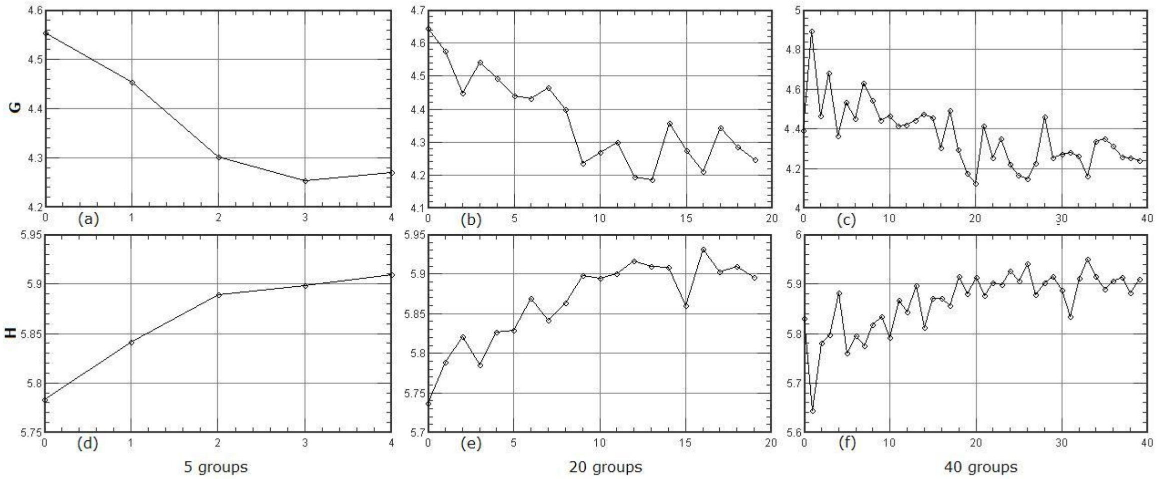

Additional graphs for Fig. 3 : The 19055 genes were arranged into 5, 20 and 40 groups of increasing NXScores for the -1500 +500 promoter regions, and the mean tissue specificity level for each band was calculated using Grubbs' test (a, d and c) and Shannon entropy (d, e and f).
