# Supplementary material for: Prediction and analysis of nucleosome exclusion regions in the human genome
Source: BMC Genomics. 2008 Apr 22;9:186. doi: 10.1186/1471-2164-9-186 (PMC2386137; doi:10.1186/1471-2164-9-186)
Supplement: Additional file 3 — Graphs of additional genes for Figure 6. [file 1471-2164-9-186-S3.pdf]

Additional graphs for Figure 6. NXSensor results for the region -1500 to +500 from the RefSeq annotated TSS are shown as a custom track on the UCSC Genome Browser along with the RefSeq genes track. The grey bars superimposed on the NXSensor graphics denote the position of nucleosome depleted regions according to data available from Ozsolak *et al.*, 2007. A great deal of correlation is apparent, lack of correlation can be partially explained by the stringency of the nucleosome exclusion algorithm we used.

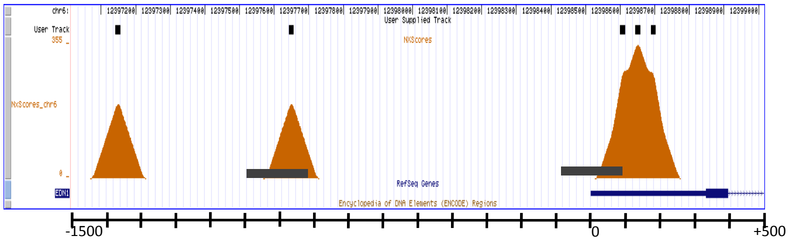

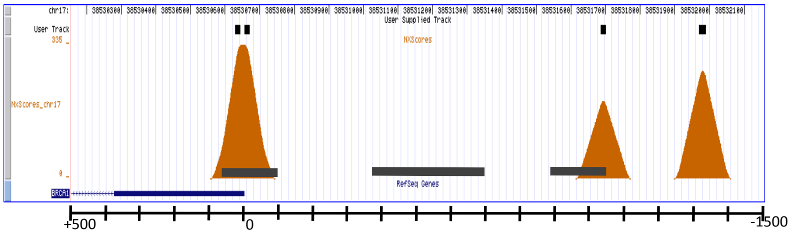

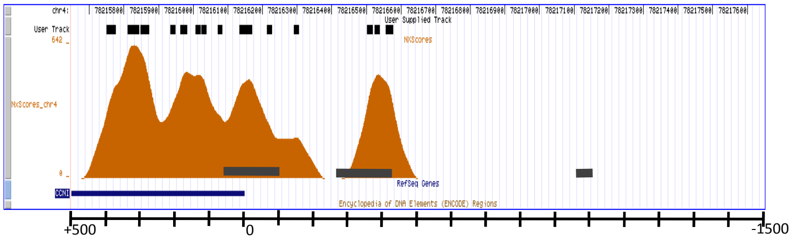

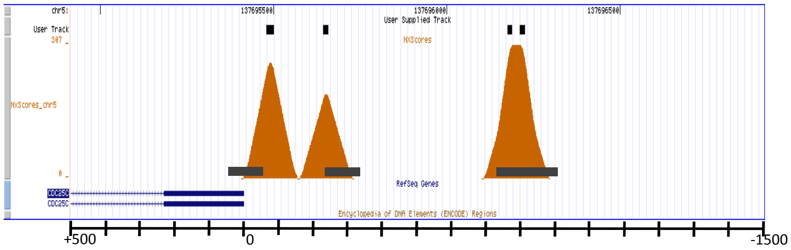

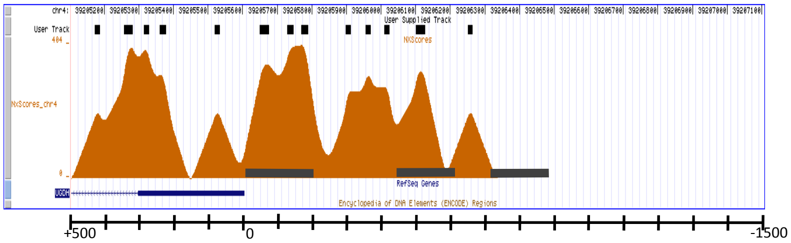

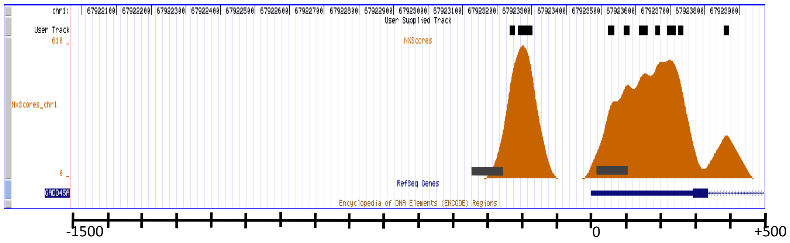

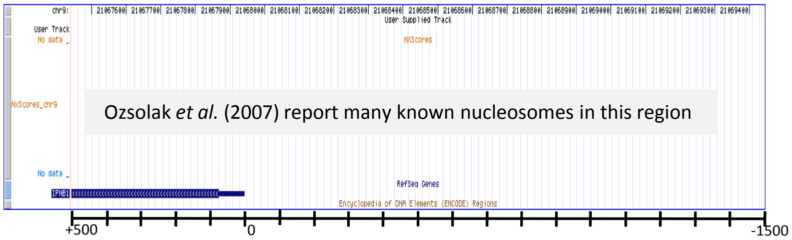
Ozsolak et al. (2007) report many known nucleosomes in this region
+500
0
-1500

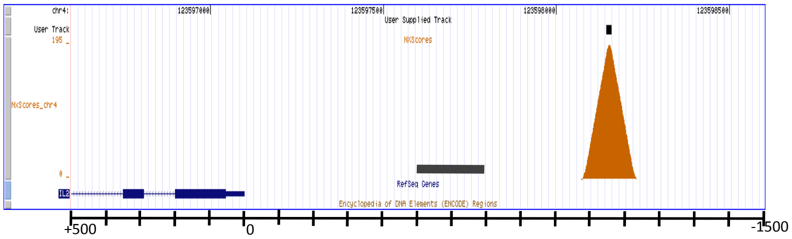
+500
0
-1500

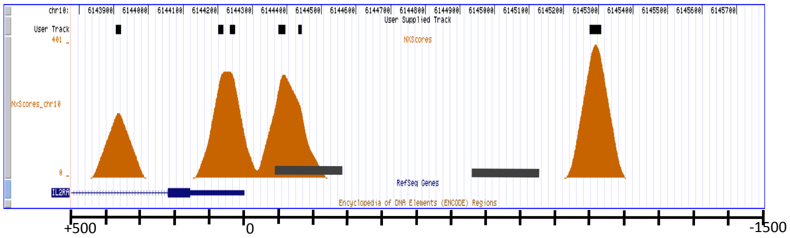
+500
0
-1500

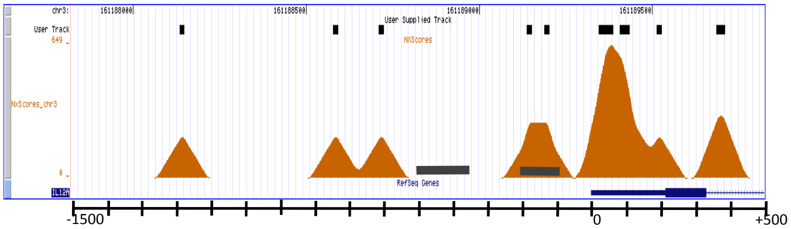
-1500
0
+500

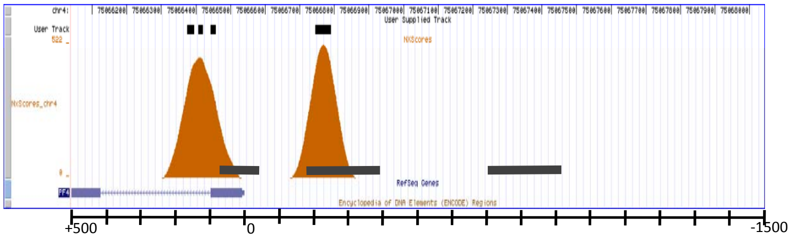
+500
0
-1500
